# Genome sequence of *Xanthomonas fuscans* subsp. *fuscans* strain 4834-R reveals that flagellar motility is not a general feature of xanthomonads

**DOI:** 10.1186/1471-2164-14-761

**Published:** 2013-11-06

**Authors:** Armelle Darrasse, Sébastien Carrère, Valérie Barbe, Tristan Boureau, Mario L Arrieta-Ortiz, Sophie Bonneau, Martial Briand, Chrystelle Brin, Stéphane Cociancich, Karine Durand, Stéphanie Fouteau, Lionel Gagnevin, Fabien Guérin, Endrick Guy, Arnaud Indiana, Ralf Koebnik, Emmanuelle Lauber, Alejandra Munoz, Laurent D Noël, Isabelle Pieretti, Stéphane Poussier, Olivier Pruvost, Isabelle Robène-Soustrade, Philippe Rott, Monique Royer, Laurana Serres-Giardi, Boris Szurek, Marie-Anne van Sluys, Valérie Verdier, Christian Vernière, Matthieu Arlat, Charles Manceau, Marie-Agnès Jacques

**Affiliations:** 1 INRA, UMR1345 Institut de Recherche en Horticulture et Semences, F-49071, Beaucouzé, France; 2 AGROCAMPUS OUEST, UMR1345 Institut de Recherche en Horticulture et Semences, F-49045, Angers, France; 3 Université d’Angers, UMR1345 Institut de Recherche en Horticulture et Semences, SFR 4207 QUASAV, PRES L’UNAM, F-49045, Angers, France; 4 INRA, LIPM UMR 441, F-31326, Castanet-Tolosan, France; 5 CNRS, LIPM UMR 2594, F-31326, Castanet-Tolosan, France; 6 CEA, Genoscope, Centre National de Séquençage, F-91057, Evry Cedex, France; 7 Universidad de Los Andes, Laboratorio de Micología y Fitopatología Uniandes, Bogotá, Colombia; 8 CIRAD, UMR BGPI, F-34398, Montpellier Cedex 5, France; 9 CIRAD, UMR PVBMT, F-97410, Saint-Pierre, La Réunion, France; 10 Université de la Réunion, UMR PVBMT, F-97715, Saint-Denis, La Réunion, France; 11 IRD, UMR RPB, F-34394, Montpellier Cedex 5, France; 12 GaTE Lab Botanica IBUSP 277 05508-900, São Paulo, SP, Brasil; 13 Université de Toulouse, Université Paul Sabatier, UMR LIPM, F-31326, Castanet-Tolosan Cedex, France; 14 current address: Department of Biology, Center for Genomics and Systems Biology, New York University, 10003, New York, NY, USA; 15 current address: ANSES, Laboratoire de Santé des végétaux, F-49044, Angers, France

**Keywords:** Seed-borne pathogen, Secretion system, Insertion sequence, Bean, Effector, Chemotaxis, Pseudogene

## Abstract

**Background:**

Xanthomonads are plant-associated bacteria responsible for diseases on economically important crops. *Xanthomonas fuscans* subsp*. fuscans* (*Xff*) is one of the causal agents of common bacterial blight of bean. In this study, the complete genome sequence of strain *Xff* 4834-R was determined and compared to other *Xanthomonas* genome sequences.

**Results:**

Comparative genomics analyses revealed core characteristics shared between *Xff* 4834-R and other xanthomonads including chemotaxis elements, two-component systems, TonB-dependent transporters, secretion systems (from T1SS to T6SS) and multiple effectors. For instance a repertoire of 29 Type 3 Effectors (T3Es) with two Transcription Activator-Like Effectors was predicted. Mobile elements were associated with major modifications in the genome structure and gene content in comparison to other *Xanthomonas* genomes. Notably, a deletion of 33 kbp affects flagellum biosynthesis in *Xff* 4834-R. The presence of a complete flagellar cluster was assessed in a collection of more than 300 strains representing different species and pathovars of *Xanthomonas*. Five percent of the tested strains presented a deletion in the flagellar cluster and were non-motile. Moreover, half of the *Xff* strains isolated from the same epidemic than 4834-R was non-motile and this ratio was conserved in the strains colonizing the next bean seed generations.

**Conclusions:**

This work describes the first genome of a *Xanthomonas* strain pathogenic on bean and reports the existence of non-motile xanthomonads belonging to different species and pathovars. Isolation of such *Xff* variants from a natural epidemic may suggest that flagellar motility is not a key function for *in planta* fitness.

## Background

Xanthomonads are plant-associated bacteria that establish neutral, commensal or pathogenic relationships with plants. Bacteria belonging to the genus *Xanthomonas* are known to be exclusively plant-associated organisms and do not colonize durably other niches. Globally, xanthomonads infect a wide range of economically important crops such as rice, banana, citrus, bean, tomato, pepper, sugarcane, and wheat. More than 124 monocotyledonous and 268 dicotyledonous plant species are hosts of xanthomonads [1,2]. The large host range of the genus strikingly contrasts with the typically narrow host range of individual strains that is restricted to one or several species of a botanical family [3]. Indeed, besides their very homogeneous phenotype, xanthomonads differ mainly by their host specificity. This is illustrated in the pathovar infrasubspecific division, which clusters bacterial strains causing similar symptoms on a same host range [4].

The common blight of bean (CBB), caused by *X. axonopodis* pv. *phaseoli* and *X. fuscans* subsp *fuscans* (*Xff*), is the most devastating bacterial disease of bean and one of the five major diseases of bean [5]. It causes significant yield loss that can exceed 40% (http://www.eppo.int/QUARANTINE/bacteria/Xanthomonas_phaseoli/XANTPH_ds.pdf). Seed quality losses impact not only bean production but also seed industry worldwide. Its wide geographical distribution is presumed to be due to an efficient seed transmission. CBB affects seed and pod production and marketability of common bean (*Phaseolus vulgaris* L.) but also lima bean (*P. lunatus* L.), tepary (*P. acutifolius* A. Gray), scarlet runner bean (*P. coccineus* L.), and several species belonging to *Vigna*[6]. Bean is a major crop all around the world; in the Americas and in Africa, bean is a staple crop and constitutes one of the main sources of protein for human (up to 60%) and animal feeding [7]. Bean was domesticated independently in Mesoamerica and in the southern Andes more than 3,000 years ago [8,9]. Low to moderate levels of CBB resistance have been identified in a few common bean genotypes from the Mesomerican gene pool, whereas no resistance has been identified in the large-seeded Andean gene pool [10]. The tepary bean possesses the highest level of resistance, whereas only low levels of resistance have been found in common and scarlet runner beans [10]. These resistances have been introgressed into common bean breeding lines but with little success into common bean cultivars of any market class [11]. To date, at least 24 different CBB resistance QTLs have been reported across all eleven linkage groups of common bean [11].

*X. axonopodis* pv. *phaseoli* and *Xff* colonizes both vascular tissues and parenchyma of their host. CBB agents survive epiphytically until favorable conditions for infection are reached [12]. These bacteria are well adapted to survive harsh phyllosphere conditions following epiphytic aggregation in biofilms [13]. Penetration through stomata is thought to lead to bacterial colonization of the mesophyll, causing leaf spots. Bacteria progression inside the host leads to the colonization of vascular tissues, but the wilting of the plant is observed only in severe cases of infection [6]. Main CBB symptoms are spots and necrosis, which appear on leaves, stems, pods and seeds. They are especially severe in tropical wet regions [6]. Bacterial ooze may be encountered especially on stems and pods, providing inoculum for secondary spread. In seeds, spots can be distributed all over the seed coat or restricted to the hilum area. Most notably, contamination occurs on plants and seeds that are symptomless, raising concerns about pathogen transmission [13,14].

Many important pathogenicity factors have been described for xanthomonads. To establish themselves successfully in host plants, xanthomonads first adhere to the plant surface, invade the intercellular space of the host tissue, acquire nutrients and counteract plant defense responses. The secretion of effectors into the extracellular milieu or directly into the host cell cytosol leads to successful host infection. The virulence factors allowing xanthomonads to complete these steps include adhesins, EPS, LPS, degradative enzymes and type three effectors (T3Es) [15]. CBB agents are known to secrete several fimbrial and non-fimbrial adhesins, some of which are involved in aggressiveness [16]. The mucoid appearance of *Xap* and *Xff* bacterial colonies is an indication of xanthan production, which is under the regulation of the diffusible factor DSF (our unpublished data). The role of the hrp-Type Three secretion System (T3SS) in infection and bacterial transmission to seed has been previously demonstrated [17]. A specific repertoire of 12 to 19 T3Es per strain of *Xap* and *Xff* strains has been determined [18]. However, a comprehensive characterization of all virulence factors in CBB agents remains to be proposed, and the genome deciphering of *Xff* and *Xap* strains is a first step in this direction.

CBB was first described in 1897 and the taxonomy of infecting strains is still debated since they are genetically diverse but share a common host (*Phaseolus vulgaris*) on which they induce the same range of symptoms. Among these strains, some produce a brown pigment on tyrosine-containing medium, therefore are called fuscous strains. The pigment results from the secretion and oxidation of homogentisic acid (2,5 dihydroxyphenyl acetic acid), an intermediate in the tyrosine catabolic pathway [19]. These strains are referred to as variant *fuscans* and are usually highly aggressive on bean [20,21] although the pigment itself has not been directly associated with pathogenicity [22,23]. Up to 1995, fuscous and non-fuscous strains responsible for CBB were grouped in a single taxon, namely, *X. campestris* pv. *phaseoli*. Genetic diversity of strains responsible for CBB was demonstrated by rep-PCR [24], AFLP [25] and recently by MLSA [26]. Three genetic lineages (GL2, GL3 and GL*fuscans*) are phylogenetically closely related and belong to rep-PCR group 6 [27] while GL1 is phylogenetically distant and belongs to rep-PCR group 4 [25,26]. Following taxonomical revision of the *Xanthomonas* genus, this pathovar was transferred to *X. axonopodis*, fuscous strains forming a variant within this pathovar [2,3]. The current taxonomically valid nomenclature for the strains responsible for CBB is *Xanthomonas fuscans* subsp. *fuscans* (*Xff*) for the fuscous strains, and *Xanthomonas axonopodis* pv. *phaseoli* for the non-fuscous strains [28]. Fuscous strains were first isolated by Burkholder from beans grown in Switzerland in 1924 [29] and have been isolated from every bean production area throughout the world since this date. The strain 4834-R is a highly aggressive strain that was isolated from a seed-borne epidemic in France in 1998 [13].

Twelve complete genome sequences of *Xanthomonas* are currently available and more than 90 draft *Xanthomonas* genomes (http://www.xanthomonas.org/genomes.html) are deposited in public databases. Altogether, genomes are available for strains representing 13 pathovars spanning over 11 *Xanthomonas* species. Most of the sequenced strains are pathogenic to five plant taxa (cabbage, cassava, citrus, rice, and sugarcane). No complete fully assembled genome sequence is yet available for any xanthomonads pathogenic to legumes. However the draft sequence of *X. axonopodis* pv. *glycines* strain 12–2, a pathogen of soybean, was recently made available (accession number: AJJO00000000). Common characteristics of previously released *Xanthomonas* genomes are to hold a great number of genes encoding proteins devoted to plant environment recognition such as methyl-accepting chemotaxis protein (MCP) and other sensors, to plant substrates exploitation such as TonB-dependent transporters (TBDT) and cell wall-degrading enzymes (CWDE), and to manipulation of plant defense machinery such as T3Es [30]. These bacteria contain genes encoding the six types of protein secretion systems so far described in Gram-negative bacteria. All these γ-proteobacteria are motile by a single polar flagellum. Motility is an important feature involved in plant colonization and is often considered as a pathogenicity factor. One motif of the bacterial flagellum (flg22) is a microbial-associated molecular pattern (MAMP) recognized by a transmembrane pattern-recognition receptor (FLS2) leading to PAMP-triggered immunity (PTI) [31]. Bean is known to harbor a FLS2-like gene, which expression is regulated following fungal infection [32].

Here, we provide the first whole-genome sequence of a *Xanthomonas* pathogenic on legumes. The high quality fully assembled and manually annotated genome sequence of *X. fuscans* subsp. *fuscans* strain 4834-R (*Xff* 4834-R) reveals a strong potential for adaptation to versatile environments, which appears to be a hallmark for xanthomonads.

## Results and discussion

### *Xff* 4834-R presents the classical general features of xanthomonads genomes

**Figure 1:**
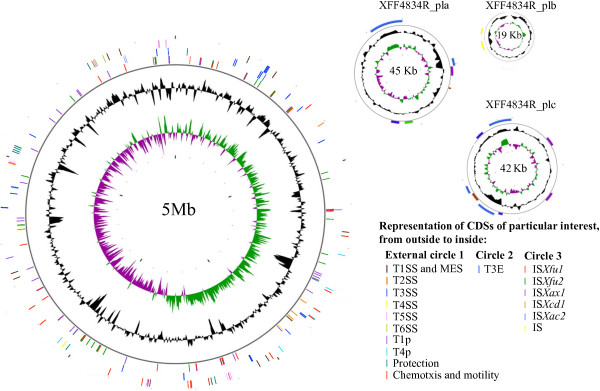
**Circular representation of the chromosome and plasmids of strain 4834-R of *X. fuscans *subsp. *fuscans*.** From outside to inside, circle 1 indicates the localization of the various secretion systems (T1SS to T6SS), type I pilus (T1p), type IV pilus (T4p), elements devoted to cell protection (exopolysaccharides, lipopolysaccharides), chemotaxis and motility. Circle 2 indicates the localization of type III effectectors (T3Es), and circle 3 indicates the localization of instertion sequences (ISs). The black circle shows the G + C content using a 100-base window. The green and purple circle shows the GC skew (G-C)/(G + C) using a 100-base window.

A high quality fully assembled sequence of the genome of *Xff* 4834-R was obtained by combining 454GS-FLX Titanium pyrosequencing (20X coverage), Illumina 36 bp (76X coverage) and Sanger (4X coverage) sequencing. The genome of *Xff* 4834-R is composed of a circular chromosome and three extrachromosomal plasmids (a, b and c) with a total size of 5 088,683 bp (Figure 1). The average GC content of *Xff* 4834-R chromosome is 64.81%, while average GC content of plasmids a, b and c are 61.32%, 60.64% and 60%, respectively. This high GC content is a common characteristic of most genera within the Xanthomonadaceae family [33]. The circular chromosome GC skew pattern is typical of prokaryotic genomes with two major shifts located near the origin and terminus of replication. The *dnaA* gene, which encodes a replication initiation factor promoting the unwinding of DNA at *oriC*, defines by convention the origin of the chromosomal sequence of *Xff* 4834-R. Annotation of the *Xff* 4834-R genome sequence revealed a total of 4,083 putative protein-coding sequences (CDSs), 137 pseudogenes, 127 insertion sequences (ISs), 54 tRNA and six rRNA genes. The rRNA genes (5S, 23S and 16S) are typically organized in two identical operons localized 463,865 bp apart. This genetic organization is a common characteristic of the other *Xanthomonas* strains sequenced (http://xanthomonas.org/genomes.html), with the exception of *X. albilineans*, which presents a reduced genome [34].

Of the 4,083 manually annotated CDSs, 3,021 have been assigned to putative functions based on homology with other known proteins and functional domain analyses (Table 1). Overall, automatic identification of clusters of orthologous groups of proteins (COGs) did not reveal any major difference in functions predicted in *Xff* 4834-R genome compared to the genomes of other *Xanthomonas* sp. (data not shown).

**Table 1:** **Putative functions assigned to *Xff *** **4834-R CDSs according to Riley classification **[35]

**Main functional class**^**1**^	**Gene ontology number (GO)**	**Number of CDSs**
1. Metabolism	GO:0008152	1,248
*1.1. Carbon compounds utilization*		*142*
*1.2. Macromolecules degradation*	*GO:0009057*	*151*
*1.3 Energy metabolism (carbon)*	*GO:0015980*	*83*
*1.4 Energy production/transport*		*57*
*1.5. Building block biosynthesis*		*260*
*1.6. Macromolecules biosynthesis*	*GO:0009059*	*214*
*1.7. Central intermediary metabolism*		*82*
*1.8. Metabolism of other compounds*		*20*
2. Information transfer		371
3. Regulation	GO:0050789	360
4. Transport	GO:0005215, GO:0006810	489
5. Cell processes	GO:0009987	186
6. Cell structure	GO:0005575	34
7. Location of gene products	GO:0005575	199
8. Extrachromosomal		136
9. DNA sites		
10. Cryptic genes		1,060

^1^ Only one putative functional class was assigned per CDS.

### Xanthomonads pan-genome and comparative genomics

The pan-genome of a bacterial genus, species or group of strains is composed of a core genome (genes shared by all individuals) and a dispensable genome consisting of partially shared and strain-specific genes [36]. The dispensable fraction reflects the diversity of the group and may contain genes involved in the diversity of lifestyles [37], *Xanthomonas* pathogenicity, and adaptation to host and tissues [30,38,39]. Based on the phylogeny of the *Xanthomonas* genus [40] and the quality of the genomic sequences, we chose 12 other genomes to perform comparative genomics analyses with *Xff* 4834-R (Table 2). These strains were chosen to represent different lifestyles and different host / tissue specificities.

**Table 2:** List of genome sequences used in comparative genomics

**Accession**	**Nomenclature**	**Strain code**	**Tissue specificity**	**Host**	**Reference**
PRJNA58657	*Stenotrophomonas maltophilia* (*Sm*)	R551-3	Non-pathogenic endophyte	Poplar	Unpublished
PRJNA43163	*Xanthomonas albilineans* (*Xal*)	GPE PC73	Vascular pathogen	Sugarcane	[34]
PRJNA73179	*X. axonopodis* subsp. *citrumelonis* (*Xacm*)	F1	Non-vascular pathogen	Citrus	[41]
PRJNA57887	*X. campestris* pv. *campestris* (*Xcc*)	ATCC33913	Vascular pathogen	Brassicacaeae	[42]
PRJNA55437	*X. campestris* pv. *musacearum* (*Xcm*)	NCPPB4381	Vascular pathogen	Banana	[43]
PRJNA159539	*X. campestris* pv. *raphani* (*Xcr*)	756C	Non-vascular pathogen	*Brassica* spp.	[39]
PRJNA57889	*X. citri* subsp. *citri* (*Xac*)	306	Non-vascular pathogen	Citrus	[42]
PRJNA58321	*X. euvesicatoria* (*Xcv*)	85-10	Non-vascular pathogen	Tomato and sweet pepper	[44]
PRJNA47495	*X. fuscans* subsp. *aurantifolii* (*Xfa*)	ICPB10535	Non-vascular pathogen	Citrus	[45]
PRJNA63615	*X. gardneri* (*Xg*)	ATCC19865	Non-vascular pathogen	Tomato and sweet pepper	[46]
PRJNA153105	*X. oryzae* pv. *oryzae* (*Xoo*)	PXO99A	Vascular pathogen	Rice	[47]
PRJNA54411	*X. oryzae* pv. *oryzicola* (*Xoc*)	BLS256	Non-vascular pathogen	Rice	[39]
PRJNA63613	*X. vesicatoria* (*Xv*)	ATCC35937	Non-vascular pathogen	Tomato and sweet pepper	[46]
PRJNA57869	*Xylella fastidiosa* (*Xf*)	Temecula1	Vascular pathogen, insect-transmitted	Grapewine	[48]

#### More than 80% of CDSs unique to *Xff* 4834-R encodes hypothetical proteins

**Figure 2:**
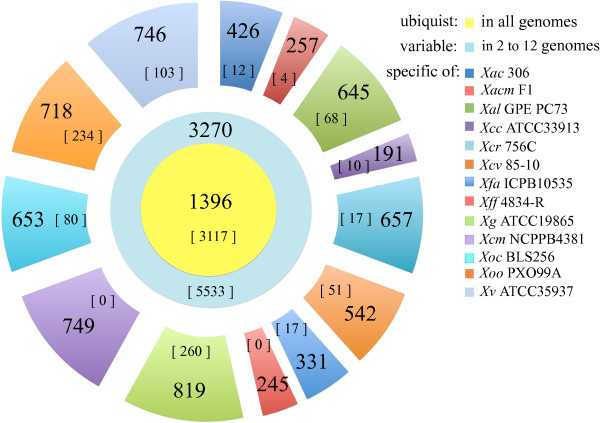
**Pan genome of 13 *Xanthomonas *sp. strains.** The 13 genome sequences (number of CDSs) included in the orthoMCL analysis are: *X. fuscans* subsp. *fuscans* strain 4834-R (*Xff* 4834-R; 4,086 CDSs), *X. fuscans* subsp. *aurantifolii* strain ICPB10535 (*Xfa* ICPB10535; 3,918 CDSs), *X. citri* subsp. *citri* strain 306 (*Xac* 306; 4,427 CDSs), *X. euvesicatoria* strain 85–10 (*Xcv* 85–10; 4,725 CDSs), *X. axonopodis* subsp. *citrumelonis* strain F1 (*Xacm* F1; 4,181 CDSs), *X. albilineans* strain GPE PC73 (*Xal* GPE PC73; 3,208 CDSs), *X. campestris* pv. *campestris* strain ATCC33913 (*Xcc* ATCC33913; 4,179 CDSs), *X. campestris* pv. *musacearum* strain NCPPB4381 (*Xcm* NCPPB4381; 4,209 CDSs), *X. campestris* pv. *raphani* strain 756C (*Xcr* 756C; 4,516 CDSs), *X. gardneri* strain ATCC19865 (*Xg* ATCC19865; 5,027 CDSs), *X. oryzae* pv. *oryzae* strain PXO99A (*Xoo* PXO99A; 4,988 CDSs), *X. oryzae* pv. *oryzicola* strain BLS256 (*Xoc* BLS256; 4,474 CDSs) and *X. vesicatoria* strain ATCC35937 (*Xv* ATCC35937; 4676 CDSs). Values represent the number of groups of orthologs, *i.e.* CDSs present in single copy in each genome, while values in brackets indicate the cumulated number of paralogs in the various genomes. The central disc corresponds to ubiquitous orthologs (present in the 13 genomes). The middle circle represents the variable part of the pan genome (orthologs present in 2 to 12 genomes), and the external circle represents the unique CDS of each genome.

The 13 genomes of *Xanthomonas* have various sizes (containing between 3,028 and 5,027 CDSs) and totalize 56,614 CDSs. This *Xanthomonas* pan-genome includes orthologs, paralogs, and CDSs that are specific of each strain (Figure 2). The core genome of the 13 *Xanthomonas* genomes contains 1,396 groups of orthologs (18,148 CDSs), which are defined as copy-unique genes present in every genome and also 195 groups of homologs (3,117 CDSs), which are conserved in all strains but have at least one in-paralog in at least one strain. The *Xanthomonas* core genome represents in average 30% of any *Xanthomonas* genome. This value is high compared to the core genome size of the highly diverse *Lactobacillus*, which represents approximately 15% of any *Lactobacillus* genome [49]. The *Xanthomonas* core genome increases to 44% of any *Xanthomonas* genome once *X. albilineans* strain GPE PC73 (*Xal* GPE PC73) is excluded of the analysis. This result is probably due to the markedly reduced genome of *Xal* GPE PC73 [34] and to its phylogenetic distance with all other *Xanthomonas* strains used in this study (Additional file 1).

The remaining CDSs (35,349) constitute the dispensable genome (3,270 groups of orthologs, 5,533 CDSs with paralogs, and 7,835 specific CDSs). The conserved fraction of the dispensable genome, *i.e*. CDSs present in 10 to 12 genomes, contains 1,591 groups of homologs (16,454 CDSs). The variable fraction, *i.e.* CDSs present in five to nine genomes, totalizes 782 groups of homologs (6,379 CDSs), whereas rare homologs, *i.e.* distributed in two to four genomes are in 1,581 groups (4,682 CDSs). Among those, pairwise comparisons of CDS contents in the sequenced genomes show a limited number of genes that are shared exclusively between two strains. As expected, the phylogenetically-closest strains share the highest number of CDSs (Additional file 1). In contrast, *Xff* 4834-R shares several CDSs with *Xal* GPE PC73. These genes have been probably acquired by horizontal gene transfert (HGT) events (Additional file 1). Indeed, most of these CDS (10/14) are located on plasmids; the others being clustered on the chromosome (cf. LPS section). At least 6,979 unique CDSs and 856 specific CDSs with paralogs constitute the strain-specific fraction of *Xanthomonas* pan-genome. The number of strain-specific genes is variable within the 13 *Xanthomonas* genomes; *Xff* 4834-R displays one of the smallest fractions of specific genes (Figure 2). The specific *Xff* 4834-R CDSs mainly encode hypothetical proteins (83.3% of the specific CDSs *vs.* 26% of the whole *Xff* 4834-R predicted proteome), a feature already observed in other comparative genomics analyses [36,50,51]. Regarding the origin of the *Xff*4834-R specific CDSs, 17.5% have a plasmidic origin and 15.9% are located in the vicinity of ISs. However, only 1.6% of *Xff*4834-R specific CDSs are associated with phage insertion. In addition, some *Xff* 4834-R specific CDSs also encode the T3E XopT, several regulators, transporters and secreted proteins (Additional file 2). However, increasing the number of *Xanthomonas* genomes in the comparison should decrease the number of *Xff* 4834-R specific CDSs observed in this study. For instance, the gene *xopT* is present in the strains *X. oryzae* pv. *oryzae* strain KACC10331 and MAFF311018, which have not been used in our analysis. Moreover, the CDSs of the specific fraction of *Xff* 4834-R may be not conserved in other *Xff-*related strains. Therefore, the prevalence of *Xff* 4834-R specific genes among large collections of strains deserves further analysis. Genomic comparisons provide candidates for further functional studies of *Xff* host colonization

**Figure 3:**
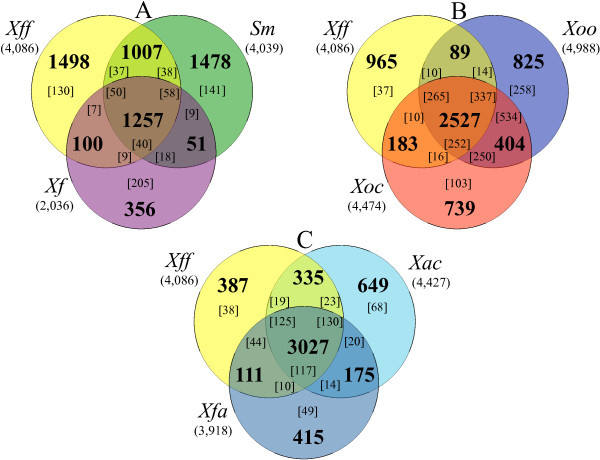
**Venn diagrams illustrating the comparisons of xanthomonads genomes.** Venn diagrams display the number of CDSs, which are present in single copy in each genome (in bold). In addition, values in brackets indicate the cumulated number of paralogs. **(A)** Comparison of the genomes of *Xanthomonas fuscans* subsp. *fuscans* strain 4834-R (*Xff*) and two distantly related strains: *Stenotrophomonas maltophilia* strain R551-3 (*Sm*), a non-pathogenic endophyte of poplar, and *Xyllella fastidiosa* strain Temecula 1 (*Xf*), an insect-vectored pathogen of grapevine. **(B)** Comparison of genomes of *Xff* 4834-R and two strains belonging to *X. oryzae*: *X. oryzae* pv. *oryzae* strain PXO99A (*Xoo*), a vacular pathogen of rice, and *X. oryzae* pv. *orizycola* strain BLS256 (*Xoc*), a non-vascular pathogen of rice. **(C)** Comparison of genomes of *Xff* 4834-R with two phylogenetically close strains: *X. axonopodis* pv. *citri* strain 306 (*Xac*), a non-vascular pathogen of citrus, and *X. fuscans* subsp. *aurantifolii* strain ICPB10535 (*Xfa*), a non-vascular pathogen of citrus.

The genome of *Xff* 4834-R was compared to different bacterial genomic sequences in order to identify functions or putative CDSs involved in plant pathogenicity and adaptation to different ecological niches (Figure 3). To get insights into functions involved in plant pathogenicity, xylem and parenchyma adaptation, the genome of *Xff* 4834-R was compared to the genomic sequences of a non-pathogenic plant endophytic isolate, *Sm* R551-3, and a xylem-limited plant pathogen, *Xf* Temecula 1. Both strains belong to the Xanthomonadaceae family. *Xf* Temecula 1 presents a reduced genome and is insect-vectored [48,52]. Orthologs shared by *Xff* 4834-R and *Xf* Temecula 1 differ significantly in Riley functional classes [35] in comparison to the whole predicted proteomes (calculated **χ**^2^ = 118.69; **χ**^2^_01[_[8]_]_ = 20.09). For instance, CDSs involved in metabolism are enriched in the orthologous fraction shared between *Xff* 4834-R and *Xf* Temecula in comparison with the whole genomes (42.2% *vs.* 30.5%, respectively). Among them, there are CDSs involved in xanthan biosynthesis and several depolymerizing carbohydrates enzymes that could be involved in plant pathogenicity and xylem colonization. Orthologs shared by *Xff* 4834-R and *Sm* R551-3 also differ significantly in Riley functional classes in comparison to the whole predicted proteomes (calculated **χ**^2^ = 100.53; **χ**^2^_01[_[8]_]_ = 20.09). CDSs involved in regulation, transport, chemotaxis and motility are enriched in the orthologous groups shared by *Xff* 4834-R and *Sm* R551-3. These functional categories are generally abundant in metabolically versatile prokaryotes capable of survival in complex and variable environmental niches, especially in nutrient-scarce environments [53]. This observation is in agreement with the ability of *Xff* to survive in the phyllopshere [13,16,17], an environment which is known to be nutrient-limited [54].

In order to identify functions putatively involved in tissue colonization*,* the genome sequence of *Xff* 4834-R was compared to the genome sequences of two rice pathogens *Xoo* PXO99A and *Xoc* BLS256. While *Xff* colonizes both the vascular system and the parenchyma of its host, *Xoo* PXO99A and *Xoc* BLS256 colonize specifically the vascular system and the parenchyma of their host, respectively. Orthologs shared by *Xff* 4834-R and *Xoc* BLS256 differ significantly in Riley functional classes in comparison to the whole predicted proteomes (calculated **χ**^2^ = 180.08; **χ**^2^_01[_[8]_]_ = 20.09). For example, CDSs involved in regulation (9 CDSs coding for two-component regulatory systems), in chemotaxis (7 MCPs), in biofilm formation (*xagBCD* and a putative filamentous adhesin CDS), and in pathogenicity (T3Es such as *xopAF* and *xopAK*) are enriched in the orthologous groups shared between *Xff* 4834-R and *Xoc* BLS256. Interstingly, XopAF and XopAK were previously suspected to be involved in tissue specificity of *Xoc* BLS256 [39]. Orthologs shared by *Xff* 4834-R and *Xoo* PXO99A differ significantly in Riley functional classes in comparison to the whole predicted proteomes (calculated **χ**^2^ = 97.88; **χ**^2^_01[_[8]_]_ = 20.09). CDSs involved in transport and of unknown function were enriched. Further analyses should give information on their putative role in *Xanthomonas* survival in the vascular system.

Comparison of *Xff* 4834-R with two citrus pathogens, *Xac* 306 and *Xfa* ICPB10535, phylogenetically closely related to *Xff* 4834-R should give insights into functions involved in host specificity and xylem adaptation since both *Xac* and *Xfa* are not vascular pathogens (Figure 3C). In this comparison, CDSs specific of *Xff* 4834-R show a distribution in Riley functional classes significantly different (calculated **χ**^2^ = 1479.39; **χ**^2^_01[_[8]_]_ = 20.09) from their distribution in the whole genome and include numerous CDSs of unknown function (67.3% of the specific CDSs compared to the 26% of the CDSs of the whole genome), several T3E encoding genes (*xopF1, xopAM, xopC1, xopF2, xopJ2*, *xopT* and *xopG*) and a pectate lyase encoding gene. CDSs present in the specific fraction of *Xff* 4834-R could play a role in host specificity, as shown by the abundance of T3Es in this fraction. Indeed, repertoires of T3Es have been already pointed out as candidate determinants of host specificity in *Xanthomonas*[18]. Further studies of CDSs specific of *Xff* will confirm if some candidates are involved in bean colonization.

### Xanthomonads core functions are conserved in *Xff* 4834-R genome

#### *Xff* 4834-R is fully equipped for sensing the environment

Bacteria mainly detect environmental signals through Methylaccepting Chemotaxis Proteins (MCPs) and sensors of Two-Component Regulatory System (TCRS). MCPs are the principal components of the chemotaxis system. These transmembrane chemoreceptors direct cell locomotion by regulating the histidine kinase CheA, which in turn communicates the information to the flagellar motor by phosphorylating its cognate response regulator CheY. In *Xanthomonas*, most MCPs are clustered in a region dedicated to chemotaxis and motility [42]. This cluster is highly similar within *Xanthomonas* with the exception of *X. albilineans*, which presents a reduced number of genes encoding MCPs [55]. Twelve MCPs are scattered on the chromosome of *Xff* 4834-R, among which eleven are also present in *Xcv* 85–10 and in most other *Xanthomonas*. The minor differences among *Xff* 4834-R and other *Xanthomonas* chemotaxis clusters concern the absence of XAC1897 and XCV1938 orthologs in *Xff* 4834-R genome.

TCRSs are major signal transduction pathways allowing bacteria to adapt to changing environmental conditions. A typical TCRS consists in a membrane-bound sensor histidine kinase (HK) that perceives external stimuli and a cognate response regulator (RR) that mediates the cellular response. Signal is transduced by successive phosphorylation reactions, as for chemotaxis [56]. The high number of TCRSs in *Xanthomonas* spp. confers to these bacteria a good adaptive potential compared to other bacteria [57-59]. *Xff* 4834-R genome is composed of 122 putative TCRSs according to their InterPro domains and using criteria defined in previous studies [58-60], including 38 transmembrane sensors, 21 sensor/regulator hybrids (Hy-HKs) and 63 RRs. The number of *Xff* 4834-R TCRSs (122) is similar to that of *Xcv* 85–10 (121) Sixty-two TCRSs correspond to pairs of sensor and cognate RR. Such an organization by pair is common in *Xanthomonas* and acquisition or loss is reported to occur for both elements at the same time, reflecting a probable process of co-evolution [58].

#### *Xff* 4834-R is fully equipped for biofilm formation and multiple stress resistance

Biofilm formation allows bacteria to resist multiple stresses and requires, at least, attachment of cells and production of exopolysaccharide matrix. These individual characteristics also participate in bacterial virulence. Type IV pilus (T4P) is known to mediate a large array of functions, including twitching motility, adhesion, microcolony formation, and virulence factors [61]. Twitching motility occurs through extension, attachment, and then retraction of the T4P. The T4P of *Xff* 4834-R is encoded by a large number of genes grouped in clusters scattered all over the genome with 24 out of 32 genes grouped in four main clusters. *Xff* 4834-R T4P belongs to T4a family, which is structurally related to type 2 secretion system (T2SS) [62]. The major pilin subunit *pilA*, as well as three *pilA-*related and one putative minor pilin subunit *pilE* genes are identified in *Xff* 4834-R genome. While synteny and identity are conserved for most genes involved in T4P synthesis among *Xanthomonas*, *pilQ* of *Xff* 4834-R is disrupted by a frameshift (Additional file 3). Since PilQ is essential for type IV pilus secretion across outer membrane [63-65], it is tempting to speculate that the T4P of *Xff* 4834-R is unfunctional. However, the truncated PilQ of *Xff* 4834-R still contained a secretin domain (IPR011662) according to Interproscan software, thus suggesting that PilQ could still be functional in *Xff* 4834-R. This is in agreement with our previous study, which show that T4P should be functional in *Xff* 4834-R [16]. Indeed, a mutant deleted in *pilA* displayed altered adhesion capacities on bean seeds relative to wild-type 4834-R, and a decreased aggressiveness. Therefore either the frameshift observed in *pilQ* has no major consequences on the protein function, or alternative secretins such as XpsD and or XcsD are recruited.

Bacterial attachment, the first step of biofilm formation, depends mainly on adhesion factors such as T4P, Type 1 pilus, and non-fimbrial adhesins. *Xff* 4834-R genome possesses a cluster of genes encoding a Type 1 pilus, belonging to γ1 fimbrial clade of the Chaperon-Usher system [66] and sometimes referred to as Type 7 secretion system [67]. This cluster (XFF4834R_chr30690- XFF4834R_chr30740) is highly similar to that of *Xac* 306 with two genes encoding the putative pili assembly chaperones, two genes encoding candidate structural fimbrial subunits containing each a spore coat U domain (IPR007893), and one gene coding a predicted usher protein, *i.e.* an outer membrane protein corresponding to the assembly platform. A conserved secreted hypothetical protein (XFF4834R_chr30730) is also predicted in this cluster as in *Xcc* ATCC33913 genome, *i.e.* between one gene coding a candidate structural fimbrial subunit and a putative pili assembly chaperone at the end of the cluster.

To date, the only identified non-fimbrial adhesins in xanthomonads are those secreted through one of the three Type 5 Secretion System (T5SS) subtypes: (i) monomeric autotransporters (T5aSS) [68], (ii) trimeric autotransporters or oligomeric coiled-coil adhesins (T5bSS) [69,70], and (iii) two-partner secretion systems including filamentous hemagglutinins (T5cSS) [71]. A total of nine adhesins potentially secreted by each of these subtypes are predicted in *Xff* 4834-R genome. The pattern of non-fibrillar adhesins encoded in *Xff* 4834-R genome is highly similar to that of *Xac* 306 [72] with two hemagglutinin-like YapH being monomeric autotransporters (encoded by XFF4834R_chr22670, XFF4834R_chr42170), two trimeric autotransporters homologous to YadA (XFF4834R_chr34400, XFF4834R_chr34420), one hemolysin called FhaC (XFF4834R_chr19440), and three filamentous hemagglutinins secreted through the two-partners pathway (XFF4834R_chr19450, XFF4834R_chr39820, XFF4834R_chr 39830). One putative hemagglutinin (XFF4834R_chr19550), highly similar to HecA, may be non-functional as a frameshift was confirmed in the C-ter part of the predicted peptide (Additional file 3). Functional evidence of the involvement in *in vitro* or *in planta* adhesion, biofilm formation, and virulence so far has been obtained for four of these non-fibrillar adhesins: YapH (XFF4834R_chr22670), XadA1 (XFF4834R_chr34400), XadA2 (XFF4834R_chr34420), and FhaB (XFF4834R_chr19450) [16]. They participate in the initial adhesion, three-dimensional structure of the biofilm and as a result, in the epiphytic fitness of the bacterium. A role of anti-virulence factor has been proposed for YapH in order to explain the higher aggressiveness of the mutant deleted of YapH in bean [16].

Exopolysaccharide (EPS) of *Xanthomonas* are mainly composed of xanthan, polymers of pentasaccharide repeating unit structures carrying at the non-reducing glucose residue a trisaccharide side-chain of varying extent of acylation [73]. Xanthan gum is the predominant component of the extracellular slime [74], a major component of the biofilm [75]. EPS are considered as determinants of disease as they induce the water-soaking in the intercellular space [76] and participate in wilt-induction for vascular pathogens [77]. Involvement in epiphytic fitness of strains belonging to various pathovars has also been demonstrated [78,79]. Xanthan is encoded by a cluster of 12 *gum* genes, *gumBCDEFGHIJKLM*[80]. In *Xff* 4834-R, the *gum* cluster (XFF4834R_chr26110 to XFF4834R_chr26220) is syntenic with those found in other *Xanthomonas* such as *Xcv* 85–10. A single nucleotide insertion in position 844 in *gumN* modifies the reading frame. In consequence, the TraB domain of the predicted protein is 60 aa truncated compared to functional orthologs in *Xanthomonas* sp. The 119 aa sequence in the C-terminal part of the predicted protein differs from those of the functional orthologs and is 59 aa longer than for other GumN predicted proteins in *Xanthomonas* sp. The gene *gumN* is also fragmented in *Xcv* 85–10 following the insertion of IS*1477*. In *Xoc* BLS256, a single base-exchange created a stop codon in the sequence resulting in two peptides (132 and 178 aa). Despite the co-transcription of *gumN* together with *gumB-gumM* operon in *X. oryzae* pv. *oryzae*[81], the role of *gumN* in xanthan biosynthesis is not demonstrated. The smooth aspect of *Xff* 4834-R colonies is consistent with a non-altered production of EPS. Pseudogenization of *gumN* had occurred independently in strains as different as *Xcv* 85–10, *Xoc* BLS256 and *Xff* 4834-R. This raises the question of the involvement of this gene product in the bacterial cycle?

Other genes, such as *xanA* (XFF4834R_chr34730) and *xanB* (XFF4834R_chr34740), also involved in xanthan biosynthesis [80,82], are present in *Xff* 4834-R as is the recently described *xagABC* operon (XFF4834R_chr34180 to XFF4834R_chr34200) [83]. Nevertheless, it should be noted that this latter cluster may not be functional in *Xff* 4834-R as the first gene of the operon, *xagA,* is pseudogenized by an early stop codon at its two third of its length. In other *Xanthomonas,* the *xagA* gene is highly similar to that found in *Xcc* 8004 [83]. The *pgaABCD* operon of *Escherichia coli* (equivalent to the *hmsHFRS* of *Yersinia pestis)* is another operon known to be involved in the synthesis of polysaccharides. Homologs of these genes are found in *Xff* 4834-R genome (XFF4834R_chr19430 to XFF4834R_chr19470) and in *Xac* 306 but not in *Xcv* 85–10 neither in *X. campestris* genomes. Both the PgaABCD of *E. coli* and the HmsHFRS of *Y. pestis* are known to be involved in the synthesis of polysaccharide adhesins required for biofilm formation [84,85]. The role of these various genes in EPS biosynthesis and pathogenicity of *Xff* 4834-R remains to be investigated.

#### The lipopolysaccharide of *Xff* 4835-R and the genomic plasticity of the O-antigen encoding genes

Lipopolysaccharide (LPS) is one of the major components of the outer membrane (OM) of Gram-negative bacteria. This essential component confers peculiar permeability barrier properties to the OM, protecting bacterial cell from many toxic compounds. LPS is also known to interact with host cells, inducing innate immunity in both plant and animal host [86]. LPS is an amphipathic molecule consisting of a hydrophobic glycolipid anchor termed lipid A, a hydrophilic polysaccharide portion in the core region and the O-antigen polysaccharide chain [87]. CDSs (*lpsJI*, *xanAB* and *ugd2*) involved in the biosynthesis of LPS precursors are clustered, except *pgi* and *galU* that are dispersed in the genome [80,88]. The cluster *rmlABCD*, which contributes also to the biosynthesis of the LPS carbohydrate precursors [80], is located downstream of *ugd2* in *Xff* 4834-R. The biosynthesis of the core-lipid A complex requires the convergent biosynthetic pathways of Kdo2-lipid A portion of LPS and of LPS outer-core involving nine and four genes, respectively [89], all present on the *Xff* 4834-R chromosome. The eight CDSs involved in the assembly and transport of LPS in Gram-negative bacteria [80,87-89] are also present in *Xff* 4834-R genome (*lptABCDEFG* and *msbA*).

The genomic plasticity associated with the O-antigen cluster is in accordance with previous comparative genomic studies [55,90] and might be due to intense diversifying selection and/or to HGT. Indeed, genes involved in O-antigen synthesis are present in a highly variable gene cluster and can be classified into three different groups: (i) O-unit-processing genes, (ii) genes involved in the synthesis of nucleotide sugars specially used as O-antigen residues, and (iii) genes encoding sugar transferases [91]. As in few strains of *E. coli*, *Xanthomonas* strains seem to process O-units via an ABC transporter pathway that involves Wzt and Wzm [90]. However, Wzt and Wzm homologs in *Xanthomonas* strains display considerable variation ranging from 23 to 92% identities at the amino acid level, which is not in accordance with the phylogenetic relationships of the strains. Furthermore, the *Xff* 4834-R genes of the O-antigen precursors *gmd* and *rmd* are only shared with *X. axonopodis* pv. *malvacearum*, *Xg* ATCC19865, *Xcc* ATCC33913 and *Xcv* 85–10. Distribution of sugar transferase genes is even more diverse in *Xanthomonas* strains. For instance, the bifunctional glycosyl transferases encoded by *wbdA1* and *wbdA2* has only true orthologs in *Xfa*, *X. axonopodis* pv. *malvacearum*, *X. citri* pv. *mangiferaeindicae* and *Xcv* 85–10. In addition, five genes (XFF4834R_chr34820 - XFF4834R_chr34860) are only shared with *X. axonopodis* pv. *malvacearum* and *Xal* GPE PC73*.* The homology of several contiguous CDSs of *Xff* 4834-R with those of *Xal* GPE PC73, which is not a closely related organism, may be indicative of an HGT event.

#### Nutrient acquisition and utilization

TonB-dependent transporters (TBDTs) are bacterial outer membrane proteins that allow active high affinity transport of large substrate molecules, among which iron-siderophore complexes, vitamin B12, and various carbohydrates [92-95]. TBDTs must interact with an inner membrane protein complex consisting of TonB, ExbB, and ExbD to get the proton motive force across the inner membrane to transport substrates [96]. The genome sequence of *Xff* 4834-R reveals a high number of TBDTs (70 including four pseudogenes and five CDSs with missing or incomplete functional domains) encoding genes. Such an overrepresentation of TBDTs is common in *Xanthomonas* sp. [93]. None of the TBDTs is specific of *Xff* 4834-R. Most *Xff* 4834-R TBDTs have orthologs in *Xac* 306 and many of them are conserved in xanthomonads, having also orthologs in *Xcc* ATCC33913. In *Xcc* ATCC33913, several TBDTs are part of CUT (Carbohydrate Utilization with TBDT) loci comprising also inner membrane transporters, degrading enzymes, and transcriptional regulators [93]. A CUT locus involved in sucrose utilization [93] is well conserved in *Xff* 4834-R. A second CUT locus, involved in the utilization of *N*-acetylglucosamine (GlcNac) containing substrates [97], is almost complete in *Xff* 4834-R, except for the TBDT encoding gene *nixC,* which is a pseudogene. However, this latter CUT system could be functional as orthologs of three other TBDT encoding genes are present, namely *nixA*, *nixB* and *nixD*. Furthermore, orthologs of two other TBDTs associated with GlcNac in *Xanthomonas* are also present in Xff4834-R genome, *naxA* and *naxB* corresponding to XFF4834R_chr14600 and XFF4834R_chr14590, respectively.

Finally, the four main CUT loci involved in plant xylan scavenging described in *Xcc* ATCC33913 are conserved in *Xff* 4834-R genome [98] The main loci involved in xylan utilization, namely *xytA*, *xylR*, *xytB* and *xylE* loci, contain genes with putative functional orthologs in *Xff* 4834-R. The only exception is an alpha-D-glucuronidase encoding gene, which is a pseudogene in *Xff* 4834-R (XFF4834R_chr41020 *agu67A*). Diversity in depolymerizing enzyme gene content within CUT loci among strains having different host range may reflect their adaptation to various host plant carbohydrates.

#### Regulation of virulence factors

DSF cell-cell signaling pathway is involved in the regulation of many virulence factors such as EPS synthesis, type III secretion, extracellular hydrolytic enzymes [99] and in the reversion of pathogen-induced stomatal closure [100]. This pathway involves a small diffusible signal factor (DSF), the DSF synthetase RpfF and a TCRS RpfC/RpfG [99]. DSF signaling is tightly linked to the intracellular second messenger cyclic dimeric GMP (c-di-GMP) [101,102]. This major gene cluster comprises nine genes in *Xcc* 8004 [103], while only eight are predicted in *Xff* 4834-R. Indeed, *rpfI* which encodes a regulatory protein in *Xcc*[99] is lacking. This is also the case in *Xcv* 85–10, while in *Xac* 306 both *rpfH* and *rpfI* are lacking [42]. *Xylella fastidiosa* shows a partial *rpf* cluster, which nevertheless plays a key role in regulation and pathogenicity [104]. Mutation of *rpfI* does not significantly reduce the virulence of *Xoo* KACC10859 [105].The *rpf* pathway is functional in *Xff* 4834-R and as expected an *rpfF* mutant shows an altered EPS production and displays rough colonies (our unpublished data).

Another diffusible signal molecule, DF, which was originally identified *in X. campestris,* was shown to be required for the production of xanthomonadin, EPS, systemic invasion, and H_2_O_2_ resistance, which are various biological processes that are crucial for bacterial survival and virulence [106,107]. DF is encoded by *xanB2*[108], a gene belonging to the xanthomonadin biosynthesis *pig* gene cluster [109], which was recently described as encoding a bifunctional chorismatase that hydrolyses chorismate into 3-hydroxybenzoic acid (3-HBA), the DF factor, and 4-HBA [110]. A *xanB2* mutant of *Xff* 4834-R presents, as expected, white colonies proving that the DF system is functional and involved in xanthomonadin production in this strain (data not shown). Biosynthesis of xanthomonadins is encoded by the *pig* cluster comprising about 20 CDSs, which may constitute part of a novel type II polyketide synthase pathway [110]. This *pig* cluster including *xanB2* is highly conserved among *Xanthomonas*[110] and *Xff* 4834-R did not depart from this rule. Gene content is highly conserved between *Xoo* PXO99A and *Xff* 4834-R, with the exception of orthologs to PXO_03724 and PXO_03725 (XFF4834R_chr40750 and XFF4834R_chr40740, respectively), which are located 133 kb away from the *pig* cluster. The yellow-pigmented colonies of *Xff* 4834-R prove that this system is functional.

#### Genes encoding the six types of protein secretion systems are conserved in *Xff* 4834-R

Gram-negative bacteria use various basic pathways to secrete proteins, among which virulence factors, and target them to the proper compartment. Type I, III, IV, and VI secretion systems (T1SS, T3SS, T4SS, and T6SS) allow translocation of unfolded proteins directly from the cytoplasm to the outside or directly into the host cell cytoplasm. Pathways that translocate polypeptides across the cytoplasmic membrane include general secretory (Sec) and twin-arginine (Tat) pathways. Type II and V secretion systems (T2SS and T5SS) allow crossing the outer membrane from the periplasm. Genes encoding these six secretion systems have been identified in xanthomonads strains so far sequenced [15,55,111].

The T1SS exports in a single step to the extracellular medium a wide range of proteins of different sizes and activities such as pore-forming hemolysins, adenylate cyclases, lipases, proteases, surface layers, and hemophores [112]. The T1SS consists in three proteins: an inner membrane ATP binding cassette (ABC) protein, a periplasmic adaptor also named membrane fusion protein (MFP), and an outer membrane (OMP) channel of the TolC family. Two sets of genes encoding an ABC transporter, a MFP, and an OMP are found in clusters in *Xff* 4834-R genome (XFF4834R_chr29870 to XFF4834R_chr29890 and XFF4834R_chr24540 to XFF4834R_chr24600), constituting two putative T1SS. Furthermore, the OMP TolC (XFF4834R_chr11840) could be associated to three other putative T1SS composed by sets of genes encoding MFP and ABC transporters (XFF4834R_chr35340 to XFF4834R_chr35370, XFF4834R_chr38590 to XFF4834R_chr38640, and XFF4834R_chr40790 to XFF4834R_chr40810). Some T1SS-secreted substrates carry a secretion signal located at the extreme C-terminus [112] and secretion involves a multistep interaction between the substrate and the ABC protein that stabilizes the assembled secretion system until the C-terminus is presented [113]. One putative substrate of T1SS (XFF4834R_chr17340) carrying 2 repetitions of the motif GGXGXDXXX is detected, while 38 other putative substrates carry only one repetition of this motif. The role of Type 1 secreted proteins in *Xff* 4834-R pathogenicity remains to be demonstrated.

**Figure 4:**
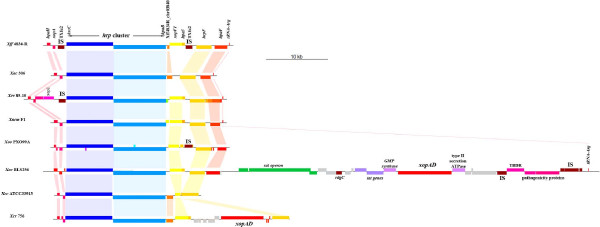
**Comparison of T3SS clusters of eight sequenced strains of *Xanthomonas*.** The organization of the *hrp* cluster encoding the T3SS and some T3-secreted proteins is compared using the R package GenoplotR for strains *Xff* 4834-R, *Xac* 306, *Xcv* 85–10, *Xacm* F1, *Xcc* ATCC33913, *Xcr* 756C, *Xoo* PXO99A, *Xoc* BLS256. Strains *Xfa* ICPB10535, *Xcm* NCPPB4381, *Xg* ATCC19865 and *Xv* ATCC35937 were not included as their *hrp*/*hrc* region is splitted on various contigs. Boxes of the same color indicate orthologous genes. Colinearity is represented by colored connectors. The *hrp* cluster is inserted in the vicinity of a *tRNA-Arg* gene, except for *Xcc* ATCC33913, *X. campestris* pv. *raphani* strain 756C (*Xcr* 756C), and *X. oryzae* pv. *oryzae* strain PXO99A (*Xoo* PXO99A). In strain *Xoc* BLS256, multiple insertions occurred between the ortholog of *hpaF* (*aka xopAF*) and the *tRNA-Arg* gene. These insertions in *Xoc* BLS256 carry virulence associated genes such as the T3E *xopAD*, TBDT, carbohydrate and salicylate esters degradation genes (*sal* operon).

Because of a similarity in the structure of these systems, Multidrug Efflux Systems (MES) are sometimes considered as T1SS [114]. MES are grouped in five families depending on the primary structure and mode of energy-coupling [115]. MES belonging to the resistance-nodulation-division (RND) and multidrug and toxic compound extrusion (MATE) families contribute significantly to intrinsic and acquired resistance to antimicrobials, but also to accommodate plant-derived antimicrobials (phytoalexins and isoflavonoids) and hence are of special interest for plant pathogens [116-119]. RND and MATE are secondary transport systems, which utilize an electrochemical gradient of cations across the membrane for drug transport. These MES consist in three components: a RND- or MATE-type exporter protein located in the cytoplasmic membrane, a gated OMP located in the outer membrane, and a MFP that links the exporter protein with the OMP. The drug transport is active and, in RND family, is driven by the proton motive force, while in MATE the drug efflux reaction is coupled with Na^+^ exchange [120]. *Xff* 4834-R genome contains seven tripartite RND-efflux pump system gene operons. Four other sets of consecutive RND exporter and the MFP coding genes could depend on *tolC* to assemble MES enabling export of drugs [112]. Two probable MATE transporters, including NorM, are identified in *Xff* 4834-R genome. In *Ralstonia solanacearum*, the RND pump AcrA and the MATE pump DinF contribute to its overall aggressiveness, probably by protecting the bacterium from the toxic effects of host antimicrobial compounds [117]. The role of these MES in *Xff* 4834-R as in other *Xanthomonas* remains to be analyzed and described. To be secreted through the T2SS and T5SS, proteins are first exported into the periplasmic space via the universal Sec or Tat pathways. The machinery of the Sec pathway recognizes a hydrophobic N-terminal leader sequence on proteins destined for secretion, and translocates proteins in an unfolded state, using ATP hydrolysis and a proton gradient for energy [121]. The *tat* and the *sec* genes are highly similar in identity and organization to those found in *Xcv* 85–10 genome. The *sec* genes are dispersed all over the genome and *secM* is absent in *Xff* 4834-R genome as it is in *Xcv* 85–10. Microsynteny and similar positions on genomes are conserved for the two T2SS *(xcs* and *xps)* identified in *Xff* 4834-R genome with orthologous clusters in *Xcv* 85–10. The T3SS encoded by the *hrp* gene cluster is a key pathogenicity factor in xanthomonads, with the exception of *X. albilineans*[55]. It is involved in the secretion and translocation of effector proteins directly into the host cell cytoplasm. In *Xff* 4834-R, the *hrp* gene cluster is inserted next to an arginine transfer-RNA (tRNA-Arg). One copy of IS*Xfu2* (see below for IS*Xfu2* description) is localized at each side of this cluster, which otherwise is almost identical and syntenic to that of other sequenced *Xanthomonas* strains (Figure 4). Genes coding the master regulators HrpG (XFF4834R_chr32700) and HrpX (XFF4834R_chr32690) are localized 3.3 Mb away from the *hrp* cluster. This type III secretion system was shown to be functional and to play a role in the colonization of bean plants and seeds [17].

T4SSs are versatile secretion systems in Gram-negative and Gram-positive bacteria that secrete a wide range of substrates, from single proteins to protein–protein and protein–DNA complexes [122-124]. Many of the T4SSs found in Gram-negative bacteria are similar to that of *Agrobacterium tumefaciens*, which comprises 12 proteins, named VirB1 to VirB11 and VirD4 [123]. T4SSs have been identified in xanthomonads and have been especially well studied in *Xac* 306 [125,126]. Two T4SS are present in *Xac* 306, one found on a plasmid and the second one on the chromosome [125]. Despite the fact that both systems belong to the same P-like T4SS group [127], the two T4SS of *Xac* 306 do not share either the same genetic organization nor high sequence identity at the protein level [125]. In *Xff* 4834-R, only the chromosomal T4SS is complete. Putative *virB5* and *virB6* are found on plasmid b and could be remnants of a plasmidic T4SS.

The T6SS is a recently characterized secretion system that appears to constitute a phage-tail-spike-like injectisome that has the potential to introduce effector proteins directly into the cytoplasm of host cells. It has been identified in many bacteria infecting plants or animals, but also in bacteria found in marine environments, the soil/rhizosphere, and in association (symbiosis, commensalism) with higher organisms [128]. In xanthomonads strains, up to two T6SS clusters have been reported. They are assigned to three different types [46]. *Xff* 4834-R contains a single T6SS belonging to the group 3, which presents a kinase/phosphatase/forkhead phosphorylation-type regulator and an AraC-type regulator. This is also the case for *X. vesicatoria*[46].

#### Xff4834-R displays a large repertoire of CWDEs

A large repertoire of T2 secreted degrading enzymes with various activities (*i.e.* protease, xylosidase, xylanase, pectate lyase, cellulase, polygalacturonase, beta-galactosidase…) is identified in *Xff* 4834-R genome. These enzymes are suspected to degrade host plant tissues. Orthologs of these 75 secreted enzymes and three pseudogenes are found in the genomes of other *Xanthomonas* sp., none seeming to be specific of *Xff* 4834-R. Orthologs of most CWDE described in Potnis *et al.*[46], or type II secretion substrates described in Szczesny *et al.*[129] are identified in *Xff* 4834-R genome. It should be noticed that no orthologs of *xynC* (XCV0965), *pel3A* and *pel10A*[46], nor of *xyn30A*, *xyl39A* and *gly43C*[98] are found in *Xff* 4834-R genome and that there are frameshifts in *agu67A* (XFF4834R_chr41020) and *xyn51A* (XFF4834R_chr41250). Interestingly, these five latter enzymes have been identified in the xylem-invading bacterium *Xcc*. The 1,4-β cellobiosidase CbhA is supposed to be required for bacteria to spread within xylem vessels [55]. While *Xff* 4834-R is known to colonize xylem vessels [6], no ortholog of *cbhA* has been found in its genome. In most xylem-invading *Xanthomonadaceae* EngXCA harbors a cellulose-binding domain (CBD) at its C-terminal extremity and a long linker region, which are known to enhance substrate accessibility [130]. *Xff* 4834-R possesses one gene encoding EngXCA (Xff4834R_chr06240), which however presents apart the CBD domain a relatively short linker domain (19 aa as in *Xac* 306). Moreover, the orthologs of *X. albilineans* genes coding CelS and XalC_0874 present neither long linker regions nor CBD in *Xff* 4834-R genome. This is also the case for other *Xanthomonas*[55]. Such differences in depolymerizing enzyme content between these two xylem-invading bacteria (*Xcc* and *X. albilineans)* and *Xff* may reflect a relatively limited ability of *Xff* to colonize xylem vessels, which is in accordance with infrequent vessel obstructions, necrosis, and wilting symptoms.

#### *Xff* 4834-R harbors a specific repertoire of putative T3Es

To mine for the presence of genes coding candidate T3Es (including T3 secreted proteins, T3SPs), we first blasted on the genome of *Xff* 4834-R the sequence of all known T3Es genes listed on the *Xanthomonas.org* website. Such a mining of the genome of *Xff* 4834-R predicts 29 genes encoding T3E orthologs (Table 3), thereby revealing a T3E repertoire larger than previously described [18]. Most genes encoding T3Es are located in the chromosome; only 5 genes encoding T3Es are plasmidic (Table 3). As well, a pseudogene similar to the 5’-end of *xopF2* and an extra pseudogenized version of *xopAD* may be found on the chromosome. Among the genes encoding T3Es found in the genome of *Xff* 4834-R, six have orthologs in all sequenced strains of *Xanthomonas* possessing an hrp-T3SS. Based on such an observation, a core effectome of the genus featuring *xopN, xopQ*, *xopF*, *xopX*, *avrBs2* and *xopP1* can be defined.

**Table 3:** **List of T3Es and T3SP identified in *Xff *** **4834-R genome and their characteristics**

**T3E / T3SP**	**Synonyms**	**Function / features**	**accession**	**GC%**	**Flanking sequences: IS, ARNt, integrase**
**Located on the chromosome**					
AvrBs2	.	Glycerophosphoryl diester phosphodiesterase	XFF4834R_chr00460	63.60	integrase
XopA	Hpa1/HpaG	"Harpin,"	XFF4834R_chr41750	60.00	IS*4*
XopAD	.	SKWP repeat protein	XFF4834R_chr40870	66.40	no
XopAE	HpaF/HpaG	LRR protein	XFF4834R_chr38990	63.60	tRNA-Arg
XopAF	AvrXv3	Unknown	XFF4834R_chr42650	49.00	transposase mutator type
XopAK	.	Unknown	XFF4834R_chr35620	58.60	no
XopAM	.	Unknown	XFF4834R_chr33550	65.30	no
XopC2	.	Haloacid dehalogenase-like hydrolase	XFF4834R_chr33300	63.20	no
XopE1	AvrXacE1	Putative transglutaminase	XFF4834R_chr02600	63.40	no
XopF1	Hpa4	Unknown	XFF4834R_chr03180	63.60	no
XopF2	.	Unknown	XFF4834R_chr18460	63.30	no
XopG	.	M27-family peptidase (Clostridium toxin)	XFF4834R_chr10930	51.10	mutator type transposase
XopI	.	F-box protein	XFF4834R_chr07620	65.10	no
XopJ5	AvrXccB	Putative C55-family cysteine protease or Ser/Thr acetyltransferase (Clan CE)	XFF4834R_chr16310	59.40	no
XopK	.	Unknown	XFF4834R_chr15450		no
XopL	XAC3090	LRR protein	XFF4834R_chr15400	61.90	no
XopN	.	ARM/HEAT repeat	XFF4834R_chr18430	63.30	no
XopP1	.	Unknown	XFF4834R_chr33320	61.60	no
XopP2	-	Unknown	XFF4834R_chr33310	60.10	no
XopQ	.	Putative inosine-uridine nucleoside N-ribohydrolase	XFF4834R_chr42130	67.50	no
XopR	.	Unknown	XFF4834R_chr25420	66.50	no
XopT	.	Unknown	XFF4834R_chr23790	64.60	IS*3*/IS*911*; transposase mutator type
XopV	.	Unknown	XFF4834R_chr42980	62.90	no
XopX	.	Unknown	XFF4834R_chr42980	65.60	no
XopZ	.	Unknown	XFF4834R_chr21120	65.50	recombination factor *rarA*
**Located on plasmids**					
XfuTAL1	"Pth, TAL"	AvrBs3/PthA-type transcription activator; 31,5 repeats of 34 aa. RVDs: NI NN NN HD NI HD HD HD HD HD NI NG NI NG NI NN NG NN HD HD NF HD NI HD HD HD HD HD NG NG	XFF4834R_plb00200	66.80	IS*3*; IS*Xac2*; Tn3 fragment; IS*Xco11*; IS*3*/IS*911*
XfuTAL2	"Pth, TAL"	AvrBs3/PthA-type transcription activator; 16,5 repeats of 34 aa. RVDs: NI NG HD NG HD NI NG NI HY NN N- HD NG HY NN HD NG	XFF4834R_pla00470		
XopC1	.	Phosphoribosyl transferase domain and haloacid dehalogenase-like hydrolase	XFF4834R_plb00200	47.80	Tn3 fragment; IS*Xco11*
XopE3	AvrXacE2	Putative transglutaminase	XFF4834R_plb00200	59.40	Tn3 fragment; IS*Xco11*

Many T3Es are located in the vicinity of various types of mobile genetic elements such as ISs or integrases in *Xff* 4834-R genome (Table 3). Interestingly, the locus carrying *xopG* contains numerous ISs on both sides of *xopG*. This locus is found in the vicinity of tRNA genes. Such genetic organization is also observed in other *Xanthomonas* genomes including *Xcv* 85–10 and *Xcc* B100. Interestingly, in the genome of *Xcc* 8004, *xopG* is pseudogenized and only one IS can be found flanking *xopG* on one side. In the genome of 4834-R, *xopG* displays a significant GC bias since the average GC content dropped to 51,1%. The predicted XopG protein belongs to the M27 family of metalloproteases. Two CDSs are located between *xopG* and *ISXfu1*. These CDSs display a GC content of 61 and 60% respectively, which remains lower than the average value in the rest of the genome (65%). The CDS XFF4834R_chr10940 encodes a putative glyoxalase that may participate in stress resistance. The CDS XFF4834R_chr10950 encodes a protein that shares structural similarity with peptidases from the M48 family. Altogether, this suggests that *xopG* is carried by a small pathogenicity island that could be transferred by HGT.

The genome of *Xff* 4834-R also features a CDS resembling the N-terminal part of *xopF2*, right upstream a complete allele of *xopF2*. Such CDS may constitute an ORPHET for terminal reassortment of novel T3Es [131]. As well, on the positive strand, CDSs Xff4834R_chr40850 and Xff4834R_chr40860 encode truncated C-terminal and N-terminal parts of XopAD, respectively. These CDSs are located right upstream a full copy of *xopAD*. The N-terminal part of XopAD features numerous repeats of a 42-residue motif identified as SKWP repeats. The N-terminal part of the full version of XopAD differs from Xff4834R_chr40860 by three indels covering five entire repeats. On the contrary, CDS Xff4834R_chr40850 shares 100% identity at the amino acid level with the C-terminal part of the full *xopAD* copy. Such an observation suggests that CDSs Xff4834R_chr40850 and Xff4834R_chr40860 constitute two functional domains that may evolve separately. The C-terminal part may then be reassorted with various N-terminal parts.

Plant-inducible promoters, also called PIP-boxes, are cis-regulatory motifs recognized by the transcriptional activator HrpX that controls the expression of T3SS and T3Es [132]. PIP boxes are located between 30 and 32 bp upstream the -10 motif of the promoter [133]. Therefore, to mine for potentially novel candidate T3Es and genes expressed in an *hrpX*-dependent manner in the genome of *Xff* 4834-R, we identified the occurrence of the previously described PIP-boxes and -10 motifs [134]. PIP-boxes matching the previously described patterns could be identified upstream *xopA*, *xopAM*, *xopAF xopE1*, *xopJ2*, *xopJ5*, *xopK*, and *xopR*. The putative PIP boxes upstream *xopA*, *xopAM*, *xopAF*, *xopJ2*, *xopJ5*, and *xopR* were located far upstream the translational start codon of the respective CDS (94 bp, 573 bp, 144 bp, 262 bp and 405 bp respectively, Additional file 4). Such an observation suggests the occurrence of very long 5’-UTRs for these genes, as already observed by Schmidtke et al. [135].

Looking at CDSs downstream putative PIP-boxes may reveal sequences corresponding to yet unidentified T3Es, as well as functions co-regulated with type III secretion (Additional file 4). Among CDS found downstream PIP boxes, CDS XFF4834R_chr23750, encoding a putative Serine/cysteine protease, could be a good candidate T3E. Genes coding for two putative polygalacturonases and a secreted lipase may be found downstreal PIP boxes, suggesting that cell wall degradation is co-regulated with type III secretion. Cell to cell bacterial communication may also be partly co-regulated with the type III secretion. Indeed, the gene *trpE* encoding a probable anthranilate synthase component is also found among genes located downstream putative PIP-boxes. The involvement of anthranilate synthases in the production of quorum signals controlling the production of virulence factors was recently documented in *Pseudomonas aeruginosa*[136].

### Eight types of insertion sequences (ISs) are present in *Xff* 4834-R genome

A total of 127 IS copies are present in the genome of *Xff* 4834-R. Among those, only 79 appear to be complete (Additional file 5) and are split into five isoforms: IS*Xax1*[137], IS*Xfu1* (https://www-is.biotoul.fr// accession number: FO203524), IS*Xfu2* (https://www-is.biotoul.fr// accession number: FO203525), IS*Xcd1* (AF263433), and IS*Xac2*[42], and three types of degenerated ISs (belonging to IS*3*-, IS*5*-, and IS*1595*- families) [137]. IS*Xfu1* has not yet been identified in any other sequenced genome but an isoform was previously sequenced (accession number: AY375317) from another bean-associated xanthomonads strain. There are 26 insertions or remnants of IS*Xfu1* found all over *Xff* 4834-R chromosome, none are plasmidic. There are 33 insertions of IS*Xfu2* in *Xff* 4834-R genome. No complete copy of IS*Xfu2* is identified so far in other xanthomonads genomes. However, exact copies of the transposase TXfu2 are present in *Xfa* ICPB10535 translated genome.

Overall, *Xff* 4834-R contains more ISs than *Xac* 306 [138] and less ISs than *X. oryzae* strains [47]. IS*Xax1* is the most abundant IS in *Xff* 4834-R genome and belongs to IS*256*-family [137]. Members of this family are plasmidic in *Xac* 306 and *Xcv* 85–10 but are present in multiple chromosomic copies in the four sequenced strains of *X. oryzae*[30]. Integration and dissemination of IS*Xax1* in *Xff* 4834-R chromosome may have occurred with the partial integration of pXCV38 plasmid (see below).

Furthermore, 12 remnants of ISs belonging to several families are also inserted in *Xff* 4834-R genome (Additional file 5). These degenerated elements are probably not functional anymore. Most remnants colocalize with other IS elements. These interdigitations of various intact or partial IS elements has been noted repeatedly in the literature [139] This may reflect the scars of consecutive but isolated transposition events resulting from selection for acquisition or loss of accessory genes.

### Occurrence of other mobile genetic elements inserted into the chromosome of *Xff* 4834-R

Several predicted viral DNA genes and fragments are found all over the genome of *Xff* 4834-R (Additional file 5). A DNA region of more than 6,500 bp contains 10 CDSs of phage-related proteins including one copy of the ϕ*Lf* filamentous phage. The CDS (XFF4834R_chr22400) coding the integrase of the ϕ*Lf* phage [140] is disrupted indicating that the protein should not be functional anymore. Two contiguous and symmetric copies of this phage are found in *Xcc* ATCC33913 genome [141]. In *Xff* 4834-R downstream of the complete ϕ*Lf* insertion, a truncated copy of ϕ*Lf* “orf112” is found contiguous to two consecutive insertions of IS*Xax1*. This suggests that IS*Xax1* insertions could be posterior to ϕ*Lf* integration and could have deleted most part of the second ϕ*Lf* integration, from which only the truncated “orf112” remained.

In addition, a chromosomal DNA region of more than 30 Kb contains CDSs that are orthologous to CDSs of plasmidic origin in other *Xanthomonas*. Half of this region (17 CDSs) is syntenic to a part of pXcB from *X. citri* pv. *aurantifolii* strain B69 [142], and 12 CDSs are syntenic to a part of pXCV38 from *Xcv* 85–10. Some CDSs of these two parts of the native plasmids are orthologous but the copies found in *Xff* 4834-R genome have higher identities with pXCV38 copies (Additional file 5), suggesting that they originate from pXCV38 rather than pXcB. It is worthwhile to mention that *pthB* from pXcB is not conserved in *Xff* 4834-R while its two adjacent CDSs are. This T3E, PthB, is required to cause cankers on citrus [142,143]. However, the gene encoding another T3E, *xopAF,* is inserted in this region together with IS*Xax1* and IS*Xfu2*. Orthologs of both *xopAF* and *Txfu2* are found by Blastp only in *Xfa* ICPB10535 genome. The association *xopAF* –IS*Xax1* is unique to *Xff* 4834-R and is not found in other xanthomonads genomes. IS*Xax1* is present in the native pXCV38 [137] and hence could have transposed from this plasmid during its integration into *Xff* 4834-R chromosome.

### Mobile genetic elements co-localize with two major chromosomal inversion events, one large DNA deletion event, and various gene insertions

Half of the IS insertion events are distributed all over the genome while the other insertions are grouped in spots of two to six ISs (Figure 1). This non-random distribution of IS elements is common in bacterial genomes [30]. IS*Xfu1* is involved in 13 IS hot spots together with IS*Xax1* and in a lesser extent with IS*Xfu2* and other IS remnants. Five other IS spots involved only IS*Xfu2*, IS*Xax1*, and IS remnants. *Xff* 4834-R ISs are associated with two major chromosomal inversion events, one large DNA deletion event, various gene transfers, and several gene breakdowns.

#### Two major chromosomal inversions co-localize with IS*Xfu1* and IS*Xfu2*

**Figure 5:**
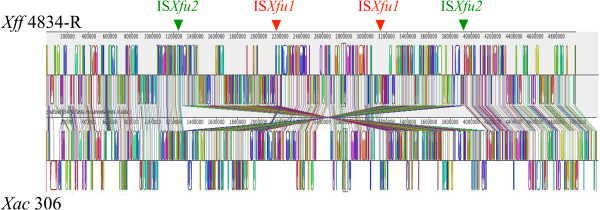
**Alignment of *Xff *4834-R and *Xac *306 genomes using Mauve.** Colored boxes and arrows indicate synthenic fractions in the genomes. Triangles correspond to ISs suspected to be involved in the inversion of two chromosomal fragments of around 1 Mb each. Green triangles are for IS*Xfu2* and red triangles for IS*Xfu1*.

A dramatic pattern of genomic rearrangement consisting in two inversion events involving IS*Xfu1* and IS*Xfu2* is revealed by comparison with the most closely related assembled genome (*Xac* 306 genome) (Figure 5). The combination of various sequencing approaches that we used ensures a high quality of the assembly and we can therefore rule out that such an inversion would originate from an error in the assembly. A considerable colinearity exists among xanthomonads genomes allowing inversion events to be easily detected, as was previously observed between *Xcc* ATCC33913 and *Xcc* 8004 [141]. Two copies of IS*Xfu1* (at positions 2,165,981 and 3,152,577) and two copies of IS*Xfu2* (at positions 1,270,755 and 3,930,499) flank the inverted segments that are located symmetrically at mirror image positions across the replication axis. Consequently, the GC skew pattern is not altered by these inversions (Figure 1). These inversions result in an inverted order of CDSs and coding strand in *Xff* 4834-R compared to the other *Xanthomonas* on the length of these two regions of around 1 Mb each (Figure 5).

#### A large deletion in the flagellar gene cluster in *Xff* 4834-R genome is associated with IS*Xfu2*

**Figure 6:**
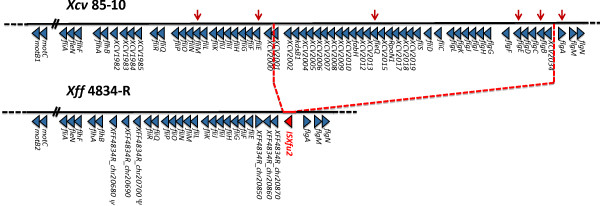
**Schematic representation of the flagellar gene clusters of *Xcv *85–10 compared to the one of *Xff *4834-R.** Position of primers used to type flagellar cluster integrity is indicated by red arrows.

**Figure 7:**
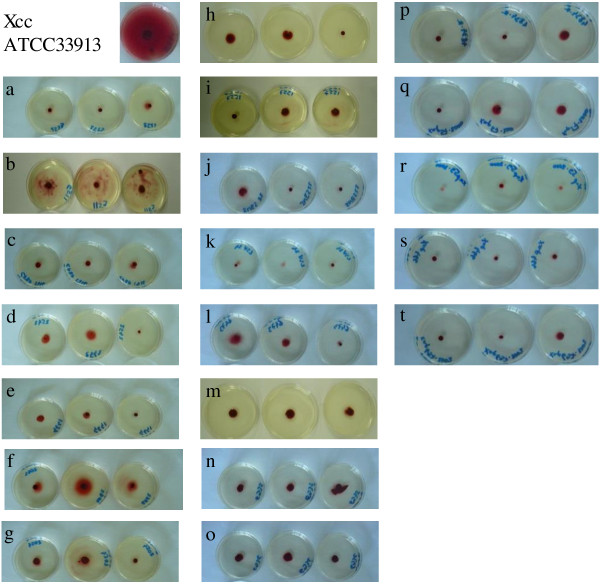
**Non-motility of some xanthomonads strains.** Illustration of non-motility as determined by soft-agar (0.2%) motility tests imaged five days after inoculation in MOKA medium containing tetrazolium chloride. The non-motile strains are **a)***X. albilineans* CFBP 2523, **b)***X. arboricola* pv. *corylina* CFBP 1159, c) *X. axonopodis* pv. *mangiferaeindicae* CFBP 1716, **d)***X. cucurbitae* CFBP 2542, **e)***X. saccharii* CFBP 4641, **f)***X. translucens* pv. *phlei* CFBP 2062, **g)***X. translucens* pv. *translucens* CFBP 2054, **h)***Xff* 4834-R, **i)** CFBP 1557, **j)** SNES 22, **k)** LSV24, **l)** CFBP 6473, **m)** CFBP 6546, **n)** CFBP 6935, **o)** CFBP 6936, **p)***Xff* 4885, **q)** Xap57, **r)** Xap59, **s)** Xap466, and **t)** Xap697. The motile *Xcc* ATCC33913 strain is used as positive controls in these tests.

Annotation of the flagellum cluster reveals that a group of 34 contiguous genes is lacking in *Xff* 4834-R genome compared to *Xcv* 85–10 genome. Instead of these genes, a complete copy of IS*Xfu2* is inserted in *Xff* 4834-R genome (Figure 6). Notably, genes coding for the periplasmic rod and its rings, the hook, and the filament are lacking. These elements are essential for flagellum biosynthesis [144]. As suspected in the absence of a functional flagellum, no swimming motility can be observed for this strain in a soft-agar assay (Figure 7). This is a surprising observation, as xanthomonads are known to be motile by means of a single polar flagellum [145]. However, as we obtained a high quality fully assembled genome, this fragment absence could not be due to sequencing errors or assembling problems. No such flagellar deletion was observed so far in any other complete assembled genome sequence of any xanthomonads. On the contrary, the flagellar cluster is highly conserved among microbes. In particular, elements such as the Flg22 peptide are usually described as canonical microbial associated molecular patterns (MAMPs) involved in the induction of the first layers of plant defense [146].

#### Absence of motility is not restricted to the strain *Xff* 4834-R and involves several species within the *Xanthomonas* genus

To determine if the event leading to a non-functional flagellum system is strain specific, pathovar specific or if, in contrast, it could be observed in other species of the genus, markers of the integrity of the flagellar cluster were searched for in several collections. To do so, seven consensus primers pairs (Additional file 6) were designed and used for PCR-amplification of genes regularly dispersed all over *Xcv* 85–10 flagellar cluster (Figure 6).

Acollection of 190 strains, mostly type strains representing most species and numerous pathovars within the *Xanthomonas* genus except CBB agents was intially used. For most strains, signals at the expected sizes were generated indicating that these strains should harbor complete flagellar cluster. However, some PCR were negative for seven strains that belong to six different species (Table 4a). Since several PCR tests were negative in each strain, this strongly indicates that one or several groups of genes could be missing. Different patterns of deletions are observed. Their impact on motility of strains was tested using soft-agar assays. None of these seven strains are motile (Figure 7a to g). Among these, the pathotype strains of *X. translucens* pv. *phlei* and *X. translucens* pv. *translucens* are not motile (Table 4 and Figure 7f and g). Moreover, the genomes of two strains belonging to *X. translucens* were recently made publicly available and also show partial or entire deletion of the flagellar cluster (Table 5), suggesting that they are also non-motile. The genome of *X. translucens* pv. *graminis* ART-Xtg29 has not one single orthologous gene (CDS with more than 80% identity on more than 80% of the length) of any gene from *Xcv* 85–10 flagellar cluster. In the genome of *X. translucens* pv. *translucens* DSM 18974, six CDSs from the flagellar cluster-I encoding protein involved in flagellar structure are lacking, thereby probably altering the motility of the strain [147]. The absence of motily could hence occur in a wide range of species or pathovars within the genus *Xanthomonas*.

**Table 4:** **Flagellar cluster integrity in a collection of 338 strains of *Xanthomonas *** **spp**

**a** ***Xanthomonas*** **collection except common bacterial blight agents**											
**Taxon**	**# or code of strains**	**Geographical origin**	**Host**	**Year of isolation**	***fliM***	***fliE***	***fleQ***	***fliC***	***flgE***	***flgB***	***flgA***
*Xanthomonas* spp.	183 strains	Diverse	Diverse	Diverse	1	1	1	1	1	1	1
*X. albilineans*	CFBP 2523	Fidgi Island	*Saccharum officinarum*	1961	1	1	1	0	0	0	0
*X. arboricola* pv. *corylina*	CFBP 1159	USA	*Corylinus maxima*	1939	0	0	0	1	1	1	1
*X. axonopodis* pv. *mangiferaeindicae*	CFBP 1716	India	*Mangifera indica*	1957	0	0	1	1	1	1	1
*X. cucurbitae*	CFBP 2542	New-Zealand	*Cucurbita maxima*	1968	1	1	0	1	0	0	0
*X. saccharii*	CFBP 4641	France	*Saccharum officinarum*	1980	1	1	0	1	0	0	0
*X. translucens* pv. *phlei*	CFBP 2062	Switzerland	*Phleum* sp.	1978	1	1	0	1	0	0	0
*X. translucens* pv. *translucens*	CFBP 2054	USA	*Hordeum vulgare*	1933	1	1	0	1	0	0	0
**b. Common bacterial blight agent collection**											
**Genetic lineage**	**# or code of strains**	**Geographical origin**	**Host**	**Year of isolation**	***fliM***	***fliE***	***fleQ***	***fliC***	***flgE***	***flgB***	***flgA***
1, 2, 3, *fuscans*	140 strains	Diverse	Diverse	Diverse	1	1	1	1	1	1	1
*fuscans*	4834-R	France	*P. vulgaris* cv. Michelet	1998	1	1	0	0	0	0	1
*fuscans*	CFBP 1557	France	*P. vulgaris* cv. Michelet	1974	1	1	0	0	0	0	1
*fuscans*	SNES 22	France	*P. vulgaris* cv. Fin de bagnols	2010	1	1	0	0	0	0	1
*fuscans*	LSV 24	France	*P. vulgaris* cv. Suisse blanc	2005	1	1	0	0	0	0	1
*fuscans*	CFBP 6473	NA^a^	NA^a^	NA	0	0	1	1	1	1	1
1	CFBP 6546	USA	*P. vulgaris*	1978	0	0	0	0	1	1	1
1	CFBP 6935	Brazil	*P. vulgaris*	1993	0	0	0	0	1	1	1
1	CFBP 6936	Brazil	*P. vulgaris*	2000	0	0	0	0	1	1	1

Strains were chosen to represent various species and pathovars within *Xanthomonas*. Signal at the expected size for each primer set (1) indicates the presence of the marker, while the absence of PCR signal at the expected size (0) is interpreted as the absence of the gene or allelic diversity and a suspected absence of motility. Absence of motility was confirmed by soft-agar motility test as illustrated in Figure 7.

^a^ Not available.

**Table 5:** **Orthologs of *Xcv *** **85–10 flagellar genes in genomes of xanthomonads**

***Xcv*** **85-10**		***Xal*** **GPE PC73**	***Xac*** **306**	***Xacm*** **F1**	***Xff*** **4834R**	***Xcc*** **ATCC 33913**	***Xcm*** **NCPPB 4381**	***Xcr*** **756c**	***Xfa*** **ICPB10535**	***Xg*** **ATCC19865**	***Xoo*** **BLS256**	***Xoo*** **PXO99a**	***Xv*** **ATCC35937**	***X. translucens*** **D61454**	***X. translucens*** **pv.** ***translucens*** **DSM 18974**	***X. translucens*** **pv.** ***graminis*** **ART-Xtg29**
**Gene**	**Product**															
XCV1977	RNA polymerase sigma factor for flagellar operon FliA	1	1	1	1	1	1	1	1	1*	1	1	1	1	1	0
XCV1978	flagellar synthesis regulator FleN	1	1	1	1	1	1*	1	1	1	1	1	1	1	1	0
XCV1979	flagellar biosynthesis regulator FlhF	1*	1	1	1	1	1	1	0	1*	1	1	1*	1	1*	0
XCV1980	flagellar biosynthesis protein FlhA	1	1	1	1	1	1	1	1	1	1	1	1	1	1	0
XCV1981	flagellar biosynthesis protein FlhB	1*	1	1	1	1	1	1	1	1	1	1	1	1	1	0
XCV1982	sensor protein	0	1	1	1*	1	1*	1	1	1	1	1	0	0	0	0
XCV1983	sensor protein	0	1	1	1	1	0	1	1	1	1	1	1	1*	0	0
XCV1984	hypothetical protein XCV1984	0	0	1	0	0	0	0	0	0	0	0	0	0	0	0
XCV1985	sensor protein	0	1	1	0	1*	1	1*	1	1*	1*	1*	1	1*	1*	0
XCV1986	flagellar biosynthesis pathway component FliR	1*	1	1	1	1	1	1	1	1	1	1	1	1	1	0
XCV1987	flagellar biosynthesis pathway component FliQ	1	1	1	1	1	1	1	0	1	1	1	1	1	1	0
XCV1988	flagellar biosynthesis protein FliP	1	1	1	1	1	1	1	1	1	1	1	1	1	1	0
XCV1989	flagellar biogenesis protein FliO	1*	1	1	1	1	1	1	0	1	1	1	1	1	1	0
XCV1990	flagellar motor switch/type III secretory pathway protein FliN	1	1	1	1	1	1	1	1	1	1	1	1	1	1	0
XCV1991	flagellar motor switch protein FliM	1	1	1	1	1	1	1	1	1	1	1	1	1	1	0
XCV1992	flagellar basal body-associated protein FliL	1*	1	1	1	1	1	1	1	1	1	1	1	1	1	0
XCV1993	flagellar hook-length control protein FliK	1*	1	0	1	1	1*	1*	1*	1	1	1	1*	1*	1*	0
XCV1994	flagellar biosynthesis chaperone FliJ	1	1	1	1	1	1	1	1	1	1	1	1	1	1	0
XCV1995	flagellar biosynthesis/type III secretory pathway ATPase FliI	1	1	1	1	1	1	1	1	1	1	1	1	1	1	0
XCV1996	flagellar biosynthesis/type III secretory pathway protein FliH	1*	1	1	1	1	1*	1	1	1	1	1	1	1	1	0
XCV1997	flagellar motor switch protein FliG	1	1	1	1	1	1	1	1	1	1	1	1	1	1	0
XCV1998	flagellar MS-ring protein	1*	1	1	1	1	1*	1	1	1	1	1	1	1	1	0
XCV1999	flagellar hook-basal body complex protein FliE	1*	1	1	1	1	1	1	1	1	1	1	1	1	1	0
XCV2000	glycosyltransferase	1*	1	1	1	1*	1*	1	1	1	1	1	1	1*	0	0
XCV2001	hypothetical protein XCV2001	0	0	0	0	0	0	0	0	0	0	0	0	0	0	0
XCV2002	hypothetical protein XCV2002	0	0	1	0	0	0	1	0	1	0	0	0	0	0	0
XCV2003	3-deoxy-manno-octulosonate cytidylyltransferase	1*	0	1	0	0	0	1	0	1	0	0	0	0	0	0
XCV2004	hypothetical protein XCV2004	0	0	1	0	0	0	1	0	1	0	0	0	0	0	0
XCV2005	hypothetical protein XCV2005	0	1*	1	0	0	0	1*	1*	1*	0	1*	1*	0	1*	0
XCV2006	hypothetical protein XCV2006	1*	1*	1	0	0	1*	1*	1*	1*	1*	1*	1*	1*	1*	0
XCV2007	Rieske 2Fe-2S family protein	1*	1*	1	0	0	1*	1*	1*	1*	1*	1*	1*	1*	1*	0
XCV2008	acetyltransferase	1*	1*	1	0	0	1*	1*	1*	1*	1*	1*	1	1*	1*	0
XCV2009	short chain dehydrogenase	1*	1*	1	0	0	1*	1*	1*	1*	1	1*	1	1*	0	0
XCV2010	short chain dehydrogenase	1	1*	1	0	0	1*	1*	1*	1*	1	1*	1	1*	1*	0
XCV2011	3-oxoacyl-ACP synthase	1	1	1	0	0	1	1*	1	1	1	1	1	1	0	0
XCV2012	acyl carrier protein	1	1	1	0	0	1	1	0	1	1	1	1	1	1	0
XCV2013	aminotransferase	1	1	1	0	1*	1	1	1	1	1	1	1	1	0	0
XCV2014	flagellar sigma-54 dependent transcriptional activator FleQ	1	1	1	0	1	1	1	1	1	1	1	1	1	1	0
XCV2015	two-component response regulator	1	1	1	0	1	1	1	1	1	1	1	1	1	0	0
XCV2016	RNA polymerase sigma-54 factor	1	1	1	0	1	1	1	1	1	1	1	1	1	1	0
XCV2017	LuxR family two-component response regulator	1	1	1	0	1	1	1	1	1	1	1		1	1	0
XCV2018	hypothetical protein XCV2018	0	1	1	0	0	1	1	1	1	1	1	0	0	1*	0
XCV2019	hypothetical protein XCV2019	0	1	1	0	0	1	1	0	1	1	1	0	0	1*	0
XCV2020	flagellin-specific chaperone FliS	1*	1	1	0	1	1	1	1	1	1	1	0	1	1	0
XCV2021	flagellar capping protein	1*	1	1	0	1	1	1	1	1	1	1	1*	1	1*	0
XCV2022	flagellin	1*	1	1	0	1	1	1	1	1	1	1	1	1	1	0
XCV2023	flagellar hook-associated protein FlgL	1*	1	1	0	1	1	1	1	1	1	1	1	1	1	0
XCV2024	flagellar hook-associated protein FlgK	1*	1	1	0	1	1*	1	1	1	1	1	1	1	1*	0
XCV2025	flagellar rod assembly protein/muramidase FlgJ	1*	1	1	0	1	1	1	1	1	1	1	1	1	1	0
XCV2026	flagellar basal body P-ring protein	1	1	1	0	1	1	1	1	1	1	1	1	1	1	0
XCV2027	flagellar basal body L-ring protein	1	1	1	0	1	1	1	1	1	1	1	1	1	0	0
XCV2028	flagellar basal body rod protein FlgG	1	1	1	0	1	1	1	1	1	1	1	1	1	1	0
XCV2029	flagellar basal body rod protein FlgF	1*	1	1	0	1	1*	1	1	1	1	1	1	1	0	0
XCV2030	flagellar hook protein FlgE	1*	1	1	0	1	1*	1	1	1	1	1	1	1	1	0
XCV2031	flagellar basal body rod modification protein	1*	1	1	0	1	1	1	1	1	1	1	1	1	0	0
XCV2032	flagellar basal body rod protein FlgC	1	1	1	0	1	1	1	1	1	1	1	1	1	1	0
XCV2033	flagellar basal-body rod protein FlgB	1	1	1	0	1	1	1	1	1	1	1	1	1	0	0
XCV2034	chemotaxis signal transduction protein	1	1	1	0	1	1	1	1	1	1	1	1	1	1	0
XCV2035	flagellar basal body P-ring biosynthesis protein FlgA	1	1	1	1	1	1	1	1	1	1	1	1	1	0	0
XCV2036	negative regulator of flagellin synthesis	1*	1	1	1	1	1	1	1	1	1	1	1	1	1	0
XCV2037	hypothetical protein XCV2037	1*	1	1	1	1	1	1	1	1	1	1	1	1	0	0

CDS are considered as orthologs (1) on the basis of an identity of more than 80% on more than 80% of the length of the CDS. CDS are considered as putative orthologs (1*) when homology is lower or on a shorter length of the CDS and if the expected functional domains are identified. On other cases, no orthologs are recorded (0).

^a^ X. translucens D61454.

^b^ X. translucens pv. translucens DSM 18974.

^c^ X. translucens pv. graminis ART-Xtg29.

Distribution of non-motile strains was also assessed in a collection of 148 strains representing the four genetic lineages (*X*. *axonopodis* pv. *phaseoli* GL1, GL2, GL3, and *Xff*) of the agents responsible for the common bacterial blight of bean. While 95% of the strains harbor a complete flagellar cluster, eight strains possess an incomplete flagellar cluster (Table 4b). Three patterns of deletion were identified. One pattern is found in several strains from the GL1 isolated in the Americas over a large period of time and another pattern is found in fuscous strains isolated from different places in France over a period of 30 years. Absence of motility was, once again, confirmed by phenotyping the strains with soft-agar motility tests (Figure 7h to o).

In order to assess the prevalence of non-flagellate strains in natural environments, 12 strains isolated from the same epidemic than *Xff* 4834-R were screened for flagellar cluster integrity and motility. These strains were sampled in the same field than *Xff* 4834-R (in 1998) and in fields representing the following bean generations (seeds harvested in 1998 field sown in 2000, and seeds harvested in 2000 field plots sown in 2002). About half of the strains isolated each year is mobile, while the other half is not (Table 6, Figure 7p to t). This suggests that two populations are cohabiting in these epidemics, one being flagellate and the other not. This suggests that a non-flagellate strain may be fit in the field, at least in mixed populations with flagellate strains, as it can naturally colonize beans and be seed-transmitted over several generations.

**Table 6:** **Patterns of flagellar clusters in *X. fuscans *** **subsp. *fuscans *** **strains isolated from successive bean generations**

**Strain code**	**Year of bean cultivation**	***fliM***	***fliE***	***fleQ***	***fliC***	***flgE***	***flgB***	***flgA***
CFBP 4885	1998	1	1	0	0	0	0	1
Xap57	1998	1	1	0	0	0	0	1
Xap59	1998	1	1	0	0	0	0	1
CFBP 4884	1998	1	1	1	1	1	1	1
Xap45	1998	1	1	1	1	1	1	1
Xap48	1998	1	1	1	1	1	1	1
Xap53	1998	1	1	1	1	1	1	1
Xap62	1998	1	1	1	1	1	1	1
Xap466	2000	1	1	0	0	0	0	1
Xap464	2000	1	1	1	1	1	1	1
Xap697	2002	1	1	0	0	0	0	1
Xap502	2002	1	1	1	1	1	1	1
Xap503	2002	1	1	1	1	1	1	1

Signal at the expected size for each primer set (1) indicates the presence of the marker, while the absence of PCR signal at the expected size (0) is interpreted as the absence of the gene or allelic diversity and a suspected absence of motility. Absence of motility was confirmed by soft-agar motility test as illustrated in Figure 7.

All the non-flagellate strains that lack FliC obviously also lack Flg22. Flg22 is a major MAMP that is recognized by its cognate receptor FLS2, thus activating basic host defense responses. Since natural populations of *Xff* may be composed of flagellate and aflagellate strains, the size of the population is likely to be underestimated by the plant host due to the lack of recognition of aflagellate strains. Therefore, the *Xff* population may overcome host defense and more easily invade its host. However, non-functionality of the flagellar cluster is not a frequent event in xanthomonads indicating that absence of motility could be a negative trait. Indeed, chemotaxis plays a major role in virulence of numerous pathogenic bacteria allowing bacteria to gain entry sites [148,149]. It is also likely that chemotaxis and motility play a role in fitness of bacteria outside the host, as in water for example. However, very little is known concerning any aspect of xanthomonads life outside their host. Without a functional flagellum, a bacterium cannot rely on chemotaxis to move toward attractants and away from repellants, and cannot locate and infect plant hosts in its natural niches, which could be considered as negative traits in natural environments.

#### IS*Xfu2* is flanking the *hrp* gene cluster on both sides colocalizing with T3E gene insertions

Breaks in synteny occur on both sides of the *hrp* cluster, in regions where various genes encoding candidate T3Es may be found. Interestingly, complete copies of IS*Xfu2* are located on each side of the *hrp* cluster of *Xff* 4834-R. Such a location coincides with loci displaying variations between genomes of *Xanthomonas* (Figure 4; [46]). Indeed, on one side of the *hrp* cluster, the locus located between *hpaB* and *hrpF* carries a copy of IS*Xfu2* and T3E genes: *hpa3* and *xopF1*. Neither IS*Xfu2* nor *xopF1* and *hpa3* are present in the genome of *Xac* 306. In *Xoo* Pxo99, the IS*Xo8* is located at the same locus as IS*Xfu2* in *Xff* 4834-R. In *Xoc* BLS256, this locus features a large insertion that carries carbohydrate degradation operons, virulence genes such as the T3E gene *xopAD*, *tat* genes, and transposases next to the tRNA-Arg. On the other side of the *hrp* cluster of *Xff* 4834-R, another copy of IS*Xfu2* is located between *hrcC* and *xopA*. In *Xcv* 85–10, IS*1595* is located at the same locus, as well as *xopD* and two genes coding hypothetical proteins. These observations suggest that in xanthomonads genomes, loci flanking the *hrp* cluster on both sides are prone to the insertion of mobile genetic elements carrying virulence genes, especially T3Es and behave as PAIs [150].

#### Gene rearrangements and insertions associated with mobile genetic elements

Integrons and gene cassette arrays are well known in clinical organisms in which they carry antibiotic resistance genes [151]. Integrons were also described in many bacteria colonizing diverse environments including plants, in which they are supposed to contribute to niche adaptation. Identification of chromosomal integrons in *Xanthomonas* is based on the presence of a DNA integrase (*intI*) homolog, a plausible integron-associated recombination site (*attI)*, and a gene cassette array bounded by *attC* formerly called 59-base element sites. In *Xanthomonas*, integron chromosomal insertion is located adjacent to the acid dehydratase gene, *ilvD*[152]. In contrast, the *ilvD* region in *Xff* 4834-R contains an IS hot spot (IS*Xfu2*, IS*Xfu1*, and IS*Xax1*) and several genes having no orthologs in other *Xanthomonas* genomes. However, integron remnants are present elsewhere in *Xff* 4834-R genome (Additional file 7). A truncated copy of *intI* is found 2.5 Mb away in *Xff* 4834-R genome, and adjacent to *intI*, the *attI* site flanked by an array of gene cassettes consisting of a single CDS and its *attC* site [152] In *Xcc* ATCC33913 integron, several copies of *pigH* are present in the cassette array. One pseudogenized copy of *pigH* is also present in the cassette array of *Xff* 4834-R. The four other cassettes of this array contain genes encoding cryptic hypothetical proteins. Contiguous to this region, a spot of 4 ISs (IS*Xax1*, IS*Xcd1,* and two copies of IS*Xfu2*) may be involved in the genomic reorganization of *ilvD* region explaining the different *Xff* 4834-R integron localizations in comparison to all other sequenced *Xanthomonas*. In these two regions, different genes with low GC% and showing no or partial similarities with genes in other *Xanthomonas* are found together with genes which phylogenies do not follow organism phylogeny (data not shown). This suggests that these genes may have been acquired by IS- and/or integron- promoted HGT.

### Most gene pseudogenizations result from indel leading to frameshift and stop codons

The availability of a deep sequenced and fully assembled genome for *Xff* 4834-R and of several genomes of closely related organisms gives the opportunity to question pseudogenization. Indeed, comparative genomics is a good mean to identify pseudogenes [153]. In *Xff* 4834-R genome, the 137 events of pseudogenization observed fall into four cases (Additional file 3). First, fragments of gene for which the mechanism of pseudogenization is not visible anymore could be detected. Fourteen gene fragments initially encoding various functions result from gene erosion by comparison with functional orthologs in other xanthomonads. Among them, *ψxopF2* seems to be a truncated and degenerated copy of *xopF2*, a T3E encoding gene located downstream. A truncated copy and a complete copy of *virB6* gene are also present in the genome of *Xff* 4834-R. Two truncated copies of *virB6* are also present in *Xac* 306 that both correspond to the N-terminal part of VirB6. Such a process of gene duplication is described to precede pseudogenization or novel function acquisition in various organisms [154]. Second, numerous pseudogenizations are due to CDS disruption by ISs. Fifteen *Xff* 4834-R pseudogenes belong to this category and most of them affect genes encoding hypothetical proteins, one element of the T4p, and one small remnant of a non-fimbrial adhesin encoding gene (XFF4834R_chr19500). For one of these pseudogenes, a preceding event of gene duplication seems to have occurred, as this latter pseudogene is located downstream its putative functional copy *fhaB*_XFF4834R_chr19450_, orthologous to *fhaB*_XAC1815_. Third, a sense codon has acquired a point mutation turning it into a stop codon causing premature termination of translation. There are 27 *Xff* 4834-R pseudogenes concerned by this kind of in-frame stop. For 25 of them, a second peptide corresponding to the C-terminal part of the protein can be predicted. RNA sequencing or functional analyses would demonstrate if some of them are still functional and could correspond to the creation of novel genes by fission. Gene fission is already known in the case of modular proteins for which fragments containing functional domain fragments can still be considered as genes. This is the case for some TCRSs [58]. Fourth, most putative pseudogenizations (81 among the 137) found in *Xff* 4834-R genome correspond to frameshifted genes consecutive to a short insertion or deletion in the sequence leading to heterologous C-terminal amino acids and/or premature termination of translation (Additional file 3).

#### A frameshift in *hmgA* could lead to fuscous pigment production in *Xff* 4834-R

A case of pseudogenization by frameshift is particularly relevant in the case of *Xff* 4834-R, as it explains the abundantly described fuscous phenotype of *Xff*. Indeed, *Xff* produce a fuscous pigment due to the disruption of tyrosin catabolism. Secretion and subsequent oxidation of homogentisic acid confers this phenotype to *Xff* strains [19]. Tyrosine is catabolized as part of normal intermediary metabolism and in the breakdown of external proteins by microorganisms. In order to describe the genetic basis of this specificity of *Xff* strains compared to most other xanthomonads, we analyzed the tyrosine degradation pathway (http://biocyc.org/META/new-image?object=TYRFUMCAT-PWY). In tyrosine degradation I pathway, tyrosine is converted into fumarate through five reactions. It starts out with the transfer of its amino group to alpha-ketoglutarate by tyrosine-glutamate aminotransferase (EC 2.6.1.5). The degradation intermediate of this transaminase reaction is 4-hydroxy-phenylpyruvate, which in turn is oxidized in the presence of vitamin C to homogentisic acid. This reaction is catalyzed by 4-hydroxyphenylpyruvate dioxygenase (EC 1.13.11.27). The ring structure of homogentisate is subsequently broken and the linear C8 unit degraded in two reaction steps to fumarate and acetoacetate, one citric acid cycle intermediate and one ketone body. Based on high sequence similarity with homologs in other xanthomonads, genes of this pathway seem to be functional in the genome of *Xff* 4834-R, with the exception of *hmgA*, the gene encoding the homogentisate oxygenase. Interestingly, this gene ψXFF4834R_chr04450 appears to be non-functional in *Xff* 4834-R, due to a frameshift at position 920. Instead of a 458 residues reported for other HmgA, the protein synthetized by *Xff* 4834-R is 306 aa long. There are no functional orthologs of this gene in *Xff* 4834-R. Correction of this mutation or complementation of *Xff* 4834-R strain with a functional allele would definitely proof that in wild type *Xff* homogentisate could not be degraded in 4-maleyl-acetoactetate and accumulates giving rise to the brown diffusible pigment of *Xff.* Indeed, this acid can spontaneously oxidize and polymerize, leading to the formation of pyomelanins [155].

#### Except for pseudogenes related to HGT events, phylogeny of *Xff* 4834-R putative pseudogenes follows the phylogeny of the organisms

In order to get insights into the pseudogene evolutionary history, we compared the phylogenetic tree of every gene family having a pseudogene in *Xff* 4834-R with the phylogeny of six housekeeping genes among the 15 genomes used for comparative genomics (Additional file 1). Two pseudogenes (XFF4834R_chr25070 and XFF4834R_chr25180), located in the integron region (see above), have nucleotidic sequences more closely related to *Xcc* ATCC33913 than to *Xfa* ICPB10535 or *Xac* 306 (Additional file 8). This is consistent with HGT and also with the deleterious impact of integrons on genomes [156]. Moreover, a third pseudogene (XFF4834R_chr33800) has a phylogeny different from that of the organisms. This pseudogene of unknown function is located in the vicinity of IS*Xax1* and then could have been acquired by HGT. The phylogeny of the other pseudogenes follows the phylogeny of the housekeeping genes reflecting a probable recent pseudogenization (Additional file 8).

## Conclusions

Genomic comparisons and *Xff* 4834-R genome annotation enlighten features involved in plant pathogenicity and adaptation to different ecological niches. We identified 29 T3Es, including TALEs, depolymerizing carbohydrate enzymes, sensors of TCRS and chemotaxis, TBDT and many proteins of unknown functions that could be involved in bean adaptation, colonization of xylem and other niches, the role of which remains to be explored. The distribution of these genes in large collections of strains representing the genetic diversity of bean bacterial blight pathogens and allele sequence comparison should reveal their evolutionary history and allow the selection of candidates for further functional analyses.

While *Xff* 4834-R is well adapted to survive in the phyllopshere and to colonize seeds notably through adhesion and biofilm aggregation [13,14,16] and is highly pathogenic on bean, genome sequence analysis reveals that this strain lacks a functional flagellum. Isolation of such variants from a natural epidemic reveals that either flagellar motility is not a key function for *in planta* fitness or that some complementation occurs within the bacterial population. Mixing of flagellated and non-flagellated cells in population could also be a strategy to avoid detection by plant defense system by reducing the targets.

Finally, the sequencing and annotation of *Xff* 4834-R genome allowed the discovery of the genetic basis of the fuscous pigment production, a characteristic of all *Xff* strains. Fuscous variants belonging to separate pathovars within *Xanthomonas* are sometimes isolated. It will be interesting to test if the same genetic basis is responsible for these phenotypes that did not fixed in these populations. It shall now be feasible to replace *ψhmgA* by a functional ortholog to identify the role of this pigment in *Xff* fitness.

## Methods

### Bacterial strain

*Xff* 4834-R is a spontaneous rifamycin resistant derivative of *Xff* CFBP4834, which was isolated from an epiphytic biofilm from an asymptomatic bean leaflet (cv. Michelet) sampled in a highly infected bean field in Beaucouzé, France, in 1998. Sequenced strain 4834-R is referred to as CFBP 4885 in the French Collection of Plant Pathogenic Bacteria (http://www.angers.inra.fr/cfbp/). This strain is highly aggressive on bean (data not shown) and well adapted to bean phyllosphere [13].

### Genome sequencing, assembly and finishing

To perform the complete sequence of *Xff* 4834-R, a mix of capillary Sanger and next-generation sequencing was used. Around 20X coverage of 454 GS-FLX (Roche, http://www.roche.com) reads were added to Sanger reads, which was derived from a 10 kb insert fragment size library. This library was constructed after mechanical shearing of genomic DNA and cloning of generated inserts into plasmid pCNS (pSU18-derived). Plasmid DNAs were purified and end-sequenced (26,522 reads) by dye-terminator chemistry with ABI3730 sequencers (Applied Biosystems, Foster City, USA) leading to an approximately 4-fold coverage. The reads were assembled by Newbler (Roche) and validated via the Consed interface (http://www.phrap.org). For the finishing phases, we used primer walking of clones, PCRs and *in vitro* transposition technology (Template Generation System™ II Kit; Finnzyme, Espoo, Finland), corresponding to 634, 66 and 2,539 additional reads, respectively. Around 76-fold coverage of Illumina reads (36 bp) were mapped, using SOAP (http://soap.genomics.org.cn), for the polishing phase as it is described by Aury *et al.*[157].

The sequences reported here have been deposited in the EMBL GenBank database, and accession numbers are FO681494, FO681495, FO681496, and FO681497 for the chromosome and for the three plasmids, respectively.

### Gene prediction and annotation

Sequence analysis and annotation were performed using iANT (integrated ANnotation Tool; [158] as described for *X. albilineans*[34]. The probabilistic Markov model for coding regions used by the gene prediction software FrameD [159] was constructed with a set of CDS sequences obtained from the public databank Swiss-Prot as revealed by BLASTX analysis. The alternative matrices were built using genes first identified in ACURs (Alternative Codon Usage Regions) based on homology and taken from the *X. albilineans* annotation process [34]. The corresponding products were automatically annotated using a protocol based on HAMAP scan [160], InterPro domain annotation and BLASTp analysis. Predicted CDSs were manually annotated individually gene by gene by an international consortium of scientists with expertise on different gene functions on xanthomonads (http://www.reseau-xantho.org/reseau_xantho/). Start codon assignment was verified with special care and suggested automatic annotations were individually expertized to generate the proposed annotations. Proteins were classified according to MultiFun classification [161]. The complete annotated genetic map, search tools (SRS, BLAST), annotation, and process classification are available at http://iant.toulouse.inra.fr/.

### Genomic comparisons

In order to perform comparative genomics with *Xff* 4834-R, 12 complete *Xanthomonas* genomes publicly available at the time of this analysis were selected (Table 2). Identification of orthologous groups between genomes was achieved by orthoMCL analyses [162] with the 15 genomes, including two closely related genera (Table 2). OrthoMCL clustering analyses were performed using the following parameters: P-value Cut-off = 1 × 10^-5^; Percent Identity Cut-off = 0; Percent Match Cut-off = 80; MCL Inflation = 1.5; Maximum Weight = 316. We modified OrthoMCL analysis by inactivating the filter query sequence during the BLASTP pre-process. From results are defined unique CDSs, corresponding to CDSs present only in one copy in one genome, and groups of orthologs that correspond to CDSs present in one copy in at least two genomes. The main part of comparative analyses of genomes and figures are deduced from their distribution. Furthermore, genomes contain CDSs that are present at least in two copies (paralogs) in one or more genomes. The abundance of this kind of CDSs is variable in *Xanthomonas* genomes, from 22.1% of the CDSs in *Xoo* PXO99A to 3.61% in *Xcr* 756C, *Xff* 4834-R having 4.75%. Distribution of this kind of CDSs with paralogs is also indicated on figures and, when possible, the number of paralogs is related to the corresponding genome. Groups of homologs refer to groups of orthologs having or not paralogs.

Chromosomal rearrangements were explored using the progressive MAUVE algorithm as implemented in Mauve v2.3.1 [163].

### Phylogeny of organisms used for genomic comparison

The complete nucleotide sequences of a set of six housekeeping genes (*atpD*, *dnaK, efP, glnA, gyrB*, *rpoD*) were extracted from the 15 genomes (Table 2). Whole amino acid sequences were aligned using ClustalW with a BLOSUM protein weight matrix and transposed back to nucleotide sequence level to gain a codon-based alignment. The alignments were manually edited with Bioedit Sequence Alignment Editor Software 7.0.9.0 [164]. Sequences were concatenated following the alphabetic order of the genes using Geneious 4.8.4. A phylogenetic tree was constructed using the Maximum Likelihood method (ML). The model of evolution for the ML analysis was determined using ModelTest 3.7 in Paup. Both hierarchical likelihood ratio test (hLRT) and the standard Akaike Information criterion (AICc) were used to evaluate the model scores. Phylogenetic tree and bootstraps values were obtained using PhyML 3.0 [165]. Bootstraps analyses were done with 1000 iterations. Trees were visualized and finalized with Mega 5.03 [166].

### Pseudogenization study

Different kind of events are considered in this study: gene fragmentation detected by genomic comparison, insertion or deletion resulting in frameshift in coding regions modifying the length or the sequence of the predicted peptide, mutations resulting in an early stop codon, and insertion of IS. Degenerated transposase or phage genes are not taken into account in this study. Differences in the N-terminal part of the predicted protein (N-terminal truncated predicted peptides) are also not considered as prediction of start codons still remains to be confirmed by RNA sequencing. Frameshifts are detected with FrameD [159]. Frameshifts in the following CDSs have been confirmed by Sanger sequencing: XFF4834R_chr09400 (glucuronoxylanase), XFF4834R_chr36560 (methyl-accepting chemotaxis protein), XFF4834R_chr32500 (endo-1, 3-beta-glucanase), XFF4834R_chr32600 (xylosidase), XFF4834R_chr41020 (alpha-glucuronidase), XFF4834R_chr26090 (GumN), XFF4834R_chr19550 (adhesin-like hemagglutinin), XFF4834R_chr34200 (XagA), XFF4834R_chr12700 (PilQ), XFF4834R_chr36560 (methyl-accepting chemotaxis protein) and XFF4834R_chr41250 (endo-1,4-beta-xylanase). Primers were selected upstream and downstream of the frameshift in order to amplify a unique sequence in *Xff* 4834-R genome. Primers were validated by blast on *Xff* 4834-R genome with parameters for short queries with a minimum number of nucleotide matches of 15 nt and a maximum number of 5 mismatches. Primers were then checked *in silico* for specific and efficient gene amplifications (Amplify software version 3.1.4). Primer description is available in Additional file 9. PCRs were performed as previously described [17] and amplicons were sequenced using Sanger technology (Genoscreen, France). Presence of CDSs that are putative pseudogenes in *Xff* 4834-R genome was assessed in the 15 genomes used in genomic comparisons by BLAST of the nucleic sequences (Additional file 3). When available, nucleic sequences of the corresponding genes were used to build a Neighbor-joining (Nj) tree using Phylip 3.69 that is further drawn with Njplot 2.3. Topology of each tree was compared with the phylogeny of the organisms as represented by the ML tree with the six housekeeping genes (see above). When pseudogene phylogeny was not congruent with the phylogeny of the organisms, genomic context of the pseudogene was analyzed further to get insight into the kind of event involved in the pseudogenization.

### Design of PCR tests for analysis of flagellar cluster diversity

Consensus primer pairs were designed based on aligned flagellar clusters of *Xfa* ICPB 10535, *Xac* 306, *Xa* pv. *manihotis* CIO151, *Xcv* 85*–*10, *Xv* ATCC 35937, *X. campestris* pv. *vasculorum* NCPPB702, *Xcm* NCPPB4381, *Xcc* ATCC 33913, *Xoc* BLS256, *Xoo* KACC10331, and *Xal* GPE PC73). These primers aimed at amplifying seven genes, *fliM*, *fliE*, *fleQ*, *fliC*, *flgE*, *flgB*, and *flgA,* chosen as markers of the flagellar cluster integrity in a collection of more than 300 *Xanthomonas* strains. The list of these strains and their characteristics is available upon request. PCR assays were performed in 20-μl volumes containing 200 μM dNTP, 0.125 μM each primer (Additional file 6), 4 μl of GoTaq 5 X buffer, 0.4 U/μl of GoTaq polymerase, and 5 μl of a boiled bacterial suspension (1 × 10^7^ CFU/ml). PCR conditions were 3 min at 94°C; followed by 35 cycles of 30 s at 94°C, 30 s at annealing temperature specific of each primer pair, an elongation time adapted to amplicon size at 72°C; and ended with 10 min at 72°C. PCR amplifications were performed in duplicate for each strain.

### Motility tests

Strain motility was tested in soft-agar assays. Xanthomonad strains were grown at 28°C up to 12 days in MOKA (yeast extract 4 g/l; casamino acids 8 g/l; KH_2_PO_4_ 2 g/l; MgSO_4_.7H_2_O 0.3 g/l) medium containing 0.2% agar and 0.05% tetrazolium chloride. A drop (10 μl) of a 1 x 10^8^ cfu/ml suspension is deposited in the middle of the plate and the radius of the colony measured every two days and imaged at five days.

## Abbreviations

ACUR: Alternative codon usage region; CDS: Protein-coding sequence; Hrp: Hypersensitive response and pathogenicity; IS: Insertion sequence; MLSA: Multilocus sequence analysis; rpf: Regulation of pathogenicity factors; T1SS to T6SS: Type I to Type VI secretion systems; T3SP: Type III secreted proteins; T3Es: Type III effectors; TCRS: Two component regulatory system; CWDE: Cell wall-degrading enzyme; TBDT: TonB-dependant transporter.

## Competing interests

The authors declare that they have no competing interests.

## Authors’ contributions

AD contributed to manual annotation of the genome and analysis of the data, and drafted parts of the manuscript. SC (Carrère) performed automatic annotation of the genome and OrthoMCL analysis. VB and SF performed sequencing of the genome. TB contributed to manual annotation of the genome and analysis of the data, and drafted part of the manuscript. RK contributed to manual annotation of the genome and analysis of the data. MLAO, SB, CB, SC (Cociancich), KD, LG, FG, EL, AM, LDN, EG, IP, SP, OP, IRS, PR, MR, BS, MAVS, VV, CV and MA contributed to manual annotation of the genome and revised the manuscript. AI performed the flagellum distribution study. LSG performed OrthoMCL analysis and contributed to pseudogene analysis. CM conceived the study and revised the manuscript. MAJ conceived the study, contributed to manual annotation of the genome and analysis of the data, drafted parts of the manuscript, and coordinated the annotation project. All authors read and approved the final manuscript.

## Supplementary Material

Additional file 1 **Distribution of CDSs exclusively shared by *Xanthomonas fuscans *** **subsp. *fuscans *** **strain 4834-R (*Xff *** **4834-R) and only one of the 15 strains used in comparative genomics.** The strains, *Xff* 4834-R, *X. fuscans* subsp. *aurantifolii* strain ICPB10535 (*Xfa* ICPB10535), *X. citri* pv. *citri* strain 306 (*Xac* 306), *X. axonopodis* subsp. *citrumelonis* strain F1 (*Xacm* F1), *X. euvesicatoria* strain 85–10 (*Xcv* 85–10), *X. campestris* pv. *musacearum* strain NCPPB4381 (*Xcm* NCPPB4381), *X. oryzae* pv. *oryzae* strain PXO99A (*Xoo* PXO99A), *X. oryzae* pv. *oryzicola* strain BLS256 (*Xoc* BLS256), *X. gardneri* strain ATCC19865 (*Xg* ATCC19865), *X. vesicatoria* strain ATCC35937 (*Xv* ATCC35937), *X. campestris* pv. *campestris* strain ATCC33913 (*Xcc* ATCC33913), *X. campestris* pv. *raphani* strain 756C (*Xcr* 756C), *Xylella fastidiosa* strain Temecula1 (Xf Temecula1), *Stenotrophomonas maltophilia* strain R551-3 (*Sm* R551-3), and *X. albilineans* strain GPE PC73 (*Xal* GPE PC73), are organized according to their phylogeny represented by the Maximum Likelihood phylogenetic tree based on six housekeeping gene sequences (*atpD*, *dnaK, efP*, *glnA*, *gyrB*, *rpoD*). Bold line indicates that bootstrap value (1000 replicates) is 100, if not, bootstrap value of the branch is indicated on the tree. Branch length for *Xf* Temecula1 is 0.6 substitution per site. Click here for file

Additional file 2 **List of the 240 unique CDSs of *Xff *** **4834-R genome based on comparisons with the genomes of 12 other *Xanthomonas *** **spp. and two closely related organisms.** Click here for file

Additional file 3 **List of putative pseudogenes and gene fragments in the genome of *Xff *** **4834R.** Click here for file

Additional file 4 **Occurrences of PIP box motifs in the genome of *Xff *** **4834R.** Click here for file

Additional file 5 **Insertion of extrachromosomal elements in *Xff *** **4834R genome and related enzymes.** Click here for file

Additional file 6 Primers for amplification of selected flagellum genes. Click here for file

Additional file 7 **Comparative maps of the integron regions in *Xcc *** **ATCC33913 and *Xff *** **4834-R.** Are represented the integrase gene fragment *intI* (blue cassette), the integron-associated recombination site (*attI,* shown as a diamond), the *pigH* gene fragment (yellow cassette), several hypothetical protein encoding genes (grey cassettes), 59-base elements (be) (small square) and several insertion sequences (red cassettes). The *attI* and 59-be sites are white filled if their recombination site does not conform to the consensus sequence 5-GTTRRRY. Colored cassettes indicate orthologs. Click here for file

Additional file 8 **Examples of phylogenetic trees (Neighbor-joining) obtained for gene families having a putative pseudogene in *Xff *** **4834-R: (a) XFF4834R_chr05500 is a frameshifted gene for which two overlapping peptides could be predicted and phylogenetic tree has a topology similar to that of housekeeping genes, (b) XFF4834R_25070-25100 is disrupted by IS** ***Xax1*** **insertion (c) XFF4834R_chr25180 is a degenerated fragment of *pigH*, probably related to an integron insertion acquired from *Xcc* and (d) XFF4834R_chr33800 is a frameshifted gene for which two peptides could still be predicted.** Click here for file

Additional file 9 Primers for verification of frameshifts of selected CDSs. Click here for file
