# Supplementary material for: Genome sequence of Xanthomonas fuscans subsp. fuscans strain 4834-R reveals that flagellar motility is not a general feature of xanthomonads
Source: BMC Genomics. 2013 Nov 6;14:761. doi: 10.1186/1471-2164-14-761 (PMC3826837; doi:10.1186/1471-2164-14-761)
Supplement: Additional file 1 — Distribution of CDSs exclusively shared by Xanthomonas fuscans subsp. fuscans strain 4834-R ( Xff 4834-R) and only one of the 15 strains used in comparative genomics. The strains, Xff 4834-R, X. fuscans subsp. aurantifolii strain ICPB10535 (Xfa ICPB10535), X. citri pv. citri strain 306 (Xac 306), X. axonopodis subsp. citrumelonis strain F1 (Xacm F1), X. euvesicatoria strain 85–10 (Xcv 85–10), X. campestris pv. musacearum strain NCPPB4381 (Xcm NCPPB4381), X. oryzae pv. oryzae strain PXO99A (Xoo PXO99A), X. oryzae pv. oryzicola strain BLS256 (Xoc BLS256), X. gardneri strain ATCC19865 (Xg ATCC19865), X. vesicatoria strain ATCC35937 (Xv ATCC35937), X. campestris pv. campestris strain ATCC33913 (Xcc ATCC33913), X. campestris pv. raphani strain 756C (Xcr 756C), Xylella fastidiosa strain Temecula1 (Xf Temecula1), Stenotrophomonas maltophilia strain R551-3 (Sm R551-3), and X. albilineans strain GPE PC73 (Xal GPE PC73), are organized according to their phylogeny represented by the Maximum Likelihood phylogenetic tree based on six housekeeping gene sequences (atpD, dnaK, efP, glnA, gyrB, rpoD). Bold line indicates that bootstrap value (1000 replicates) is 100, if not, bootstrap value of the branch is indicated on the tree. Branch length for Xf Temecula1 is 0.6 substitution per site. [file 1471-2164-14-761-S1.pdf]

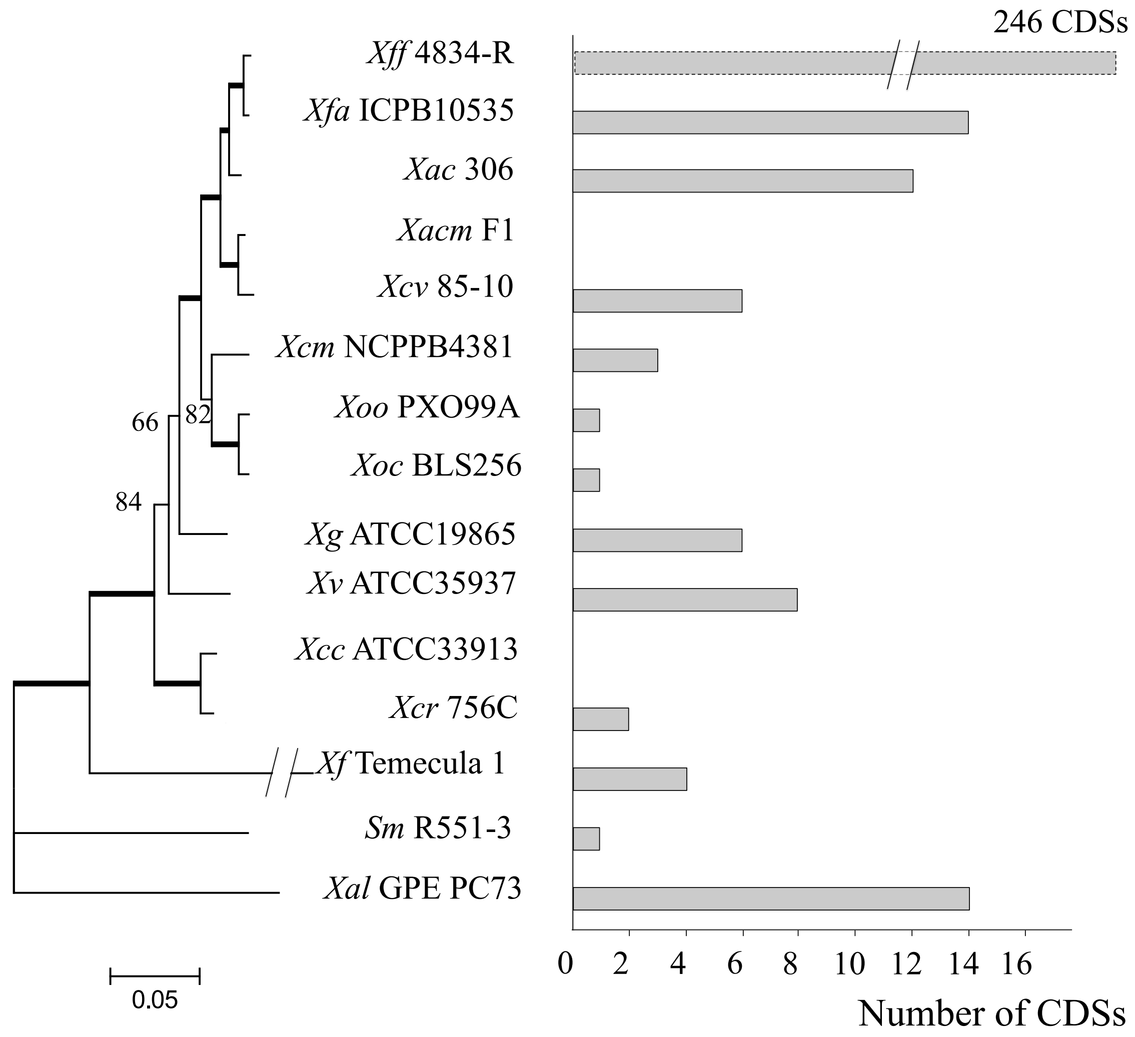

Additional file 1. Distribution of CDSs exclusively shared by *Xanthomonas fuscans* subsp. *fuscans* strain 4834-R (*Xff* 4834-R) and only one of the 15 strains used in comparative genomics. The strains, *Xff* 4834-R, *X. fuscans* subsp. *aurantifolii* strain ICPB10535 (*Xfa* ICPB10535), *X. citri* pv. *citri* strain 306 (*Xac* 306), *X. axonopodis* subsp. *citrumelonis* strain F1 (*Xacm* F1), *X. euvesicatoria* strain 85-10 (*Xcv* 85-10), *X. campestris* pv. *musacearum* strain NCPPB4381 (*Xcm* NCPPB4381), *X. oryzae* pv. *oryzae* strain PXO99A (*Xoo* PXO99A), *X. oryzae* pv. *oryzicola* strain BLS256 (*Xoc* BLS256), *X. gardneri* strain ATCC19865 (*Xg* ATCC19865), *X. vesicatoria* strain ATCC35937 (*Xv* ATCC35937), *X. campestris* pv. *campestris* strain ATCC33913 (*Xcc* ATCC33913), *X. campestris* pv. *raphani* strain 756C (*Xcr* 756C), *Xylella fastidiosa* strain Temecula1 (Xf Temecula1), *Stenotrophomonas maltophilia* strain R551-3 (*Sm* R551-3), and *X. albilineans* strain GPE PC73 (*Xal* GPE PC73), are organized according to their phylogeny represented by the Maximum Likelihood phylogenetic tree based on six housekeeping gene sequences (*atpD*, *dnaK,* *efP*, *glnA*, *gyrB*, *rpoD*). Bold line indicates that bootstrap value (1000 replicates) is 100, if not, bootstrap value of the branch is indicated on the tree. Branch length for *Xf* Temecula1 is 0.6 substitution per site.
