# Supplementary material for: Genome sequence of Xanthomonas fuscans subsp. fuscans strain 4834-R reveals that flagellar motility is not a general feature of xanthomonads
Source: BMC Genomics. 2013 Nov 6;14:761. doi: 10.1186/1471-2164-14-761 (PMC3826837; doi:10.1186/1471-2164-14-761)
Supplement: Additional file 6 — Primers for amplification of selected flagellum genes. [file 1471-2164-14-761-S6.docx]

**Additional file 6.** Primers for amplification of selected flagellum genes.

| Target | Primer code | Sequence 5' --> 3' | Tm | Fragment size (bp) |
| --- | --- | --- | --- | --- |
| *fliM* | fliM-F1 | ATCATCCGTGGGCGCATGCC | 62 | 579 |
|  | fliM-R1 | GCGGTCGCTCTGGATGCCGG |  |  |
| *fliE* | fliE-F1 | TCGCGAATCCGCAGCTACCA | 62 | 345 |
|  | fliE-R1 | TTACAGCGGCATGTTCATGA |  |  |
| *fleQ* | fleQ-F1 | TGGATGGCGGTGATGGTGGGCT | 62 | 595 |
|  | fleQ-R1 | CATCCGGCAGGCTCATGTCGCC |  |  |
| *fliC* | fliC-F1 | GCACAGGTAATCAACACCAACGTA | 58 | 1197 |
|  | fliC-R1 | TTACTGCAGCAGGCTCAGCACGTT |  |  |
| *flgE* | flgE-F1 | CGCAGCCAATGCCGATCTGAA | 62 | 594 |
|  | flgE-R1 | GTCTTGACGAAGTAGGAGGTC |  |  |
| *flgB* | flgB-F1 | ATCGCGGTGAGCATCGAGCG | 66 | 326 |
|  | flgB-R1 | CGCTTCTCGATACTGATATC |  |  |
| *flgA* | flgA-F1 | TGATCCAGGCCGGCATGATC | 58 | 338 |
|  | flgA-R1 | ATGCGCGATTCCAGGCTCAG |  |  |
