# Supplementary material for: Genome sequence of Xanthomonas fuscans subsp. fuscans strain 4834-R reveals that flagellar motility is not a general feature of xanthomonads
Source: BMC Genomics. 2013 Nov 6;14:761. doi: 10.1186/1471-2164-14-761 (PMC3826837; doi:10.1186/1471-2164-14-761)
Supplement: Additional file 7 — Comparative maps of the integron regions in Xcc ATCC33913 and Xff 4834-R. Are represented the integrase gene fragment intI (blue cassette), the integron-associated recombination site (attI, shown as a diamond), the pigH gene fragment (yellow cassette), several hypothetical protein encoding genes (grey cassettes), 59-base elements (be) (small square) and several insertion sequences (red cassettes). The attI and 59-be sites are white filled if their recombination site does not conform to the consensus sequence 5-GTTRRRY. Colored cassettes indicate orthologs. [file 1471-2164-14-761-S7.pdf]

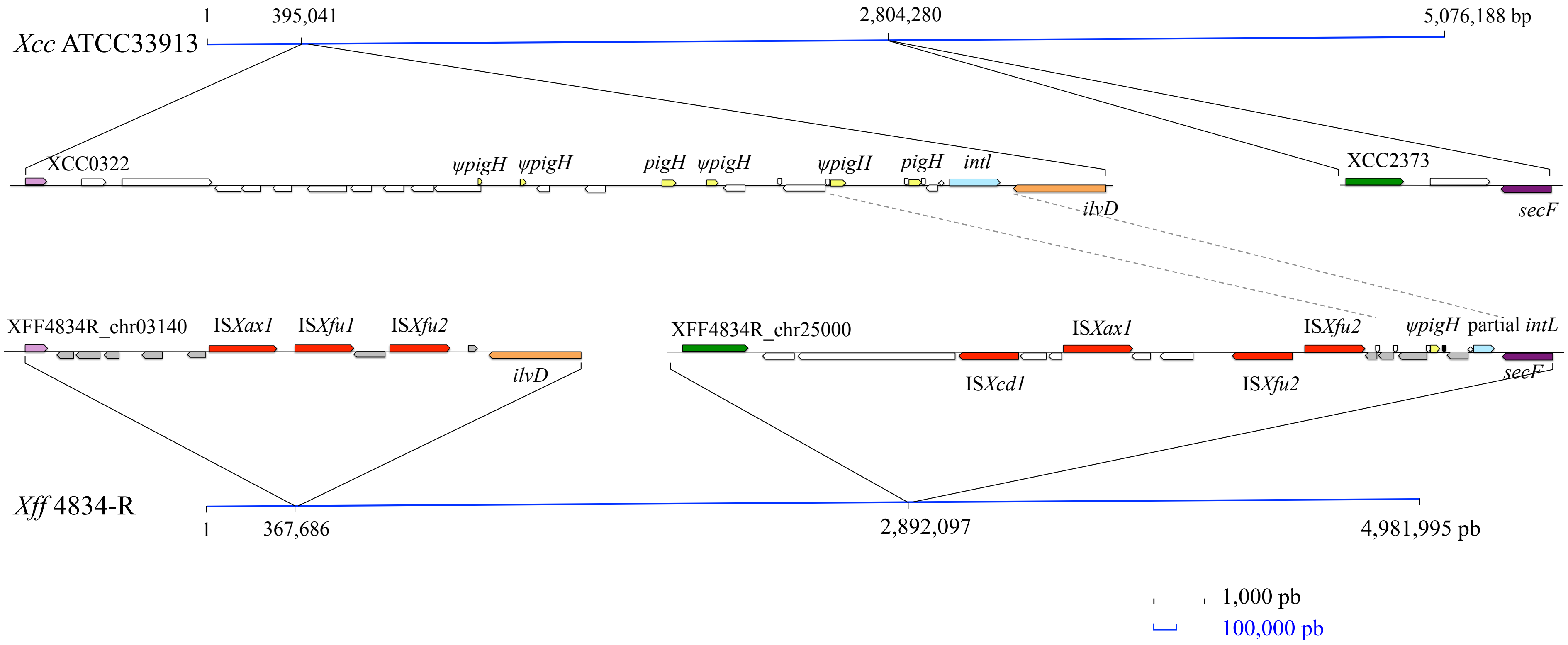

Additional file 7. Comparison of the integron regions in *Xanthomonas campestris* pv. *campestris* strain ATCC33913 (*Xcc* ATCC33913) and *X. fuscans* subsp. *fuscans* strain 4834-R (*Xff* 4834-R). Are represented the integrase gene fragment *intI* (blue cassette), the integron-associated recombination site (*attI,* shown as a diamond), the *pigH* gene fragment (yellow cassette), several hypothetical protein encoding genes (grey cassettes), 59-base elements(be) (small square) and several insertion sequences (red cassettes). The *attI* and 59-be sites are filled white if their recombination site does not conform to the consensus sequence 5-GTTRRRY. Colored cassettes indicate orthologs.
