# Supplementary material for: Genome sequence of Xanthomonas fuscans subsp. fuscans strain 4834-R reveals that flagellar motility is not a general feature of xanthomonads
Source: BMC Genomics. 2013 Nov 6;14:761. doi: 10.1186/1471-2164-14-761 (PMC3826837; doi:10.1186/1471-2164-14-761)
Supplement: Additional file 8 — Examples of phylogenetic trees (Neighbor-joining) obtained for gene families having a putative pseudogene in Xff 4834-R: (a) XFF4834R_chr05500 is a frameshifted gene for which two overlapping peptides could be predicted and phylogenetic tree has a topology similar to that of housekeeping genes, (b) XFF4834R_25070-25100 is disrupted by IS Xax1 insertion (c) XFF4834R_chr25180 is a degenerated fragment of pigH , probably related to an integron insertion acquired from Xcc and (d) XFF4834R_chr33800 is a frameshifted gene for which two peptides could still be predicted. [file 1471-2164-14-761-S8.pdf]

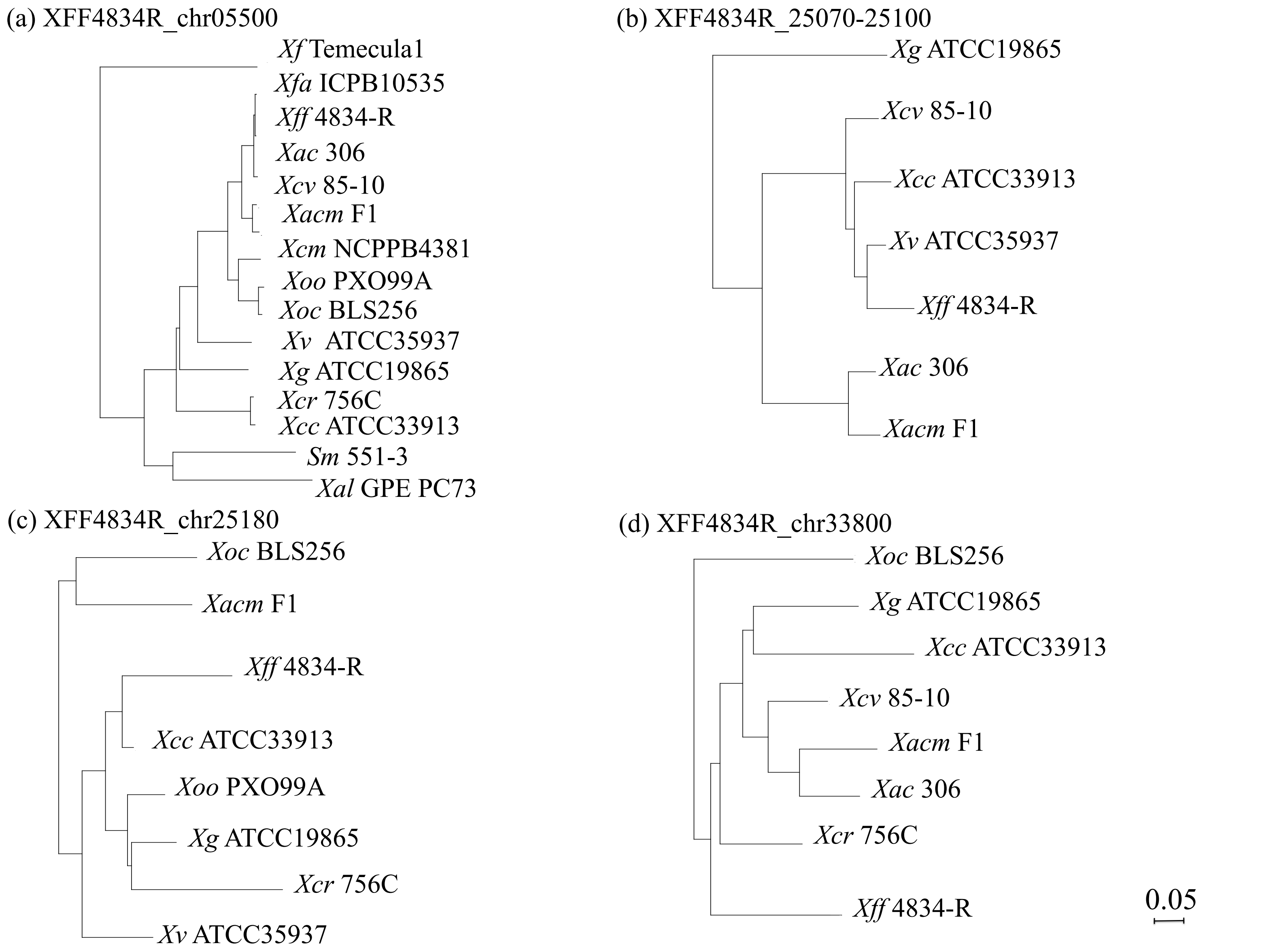

Additional file 8. Examples of phylogenetic trees (Neighbor-joining) obtained for gene families having a putative pseudogene in *Xff* 4834-R: (a) XFF4834R_chr05500 is a frameshifted gene for which two overlapping peptides could be predicted and phylogenetic tree has a topology similar to that of housekeeping genes, (b) XFF4834R_25070-25100 is disrupted by IS*Xax1* insertion (c) XFF4834R_chr25180 is a degenerated fragment of *pigH*, probably related to an integron insertion acquired from *Xcc* and (d) XFF4834R_chr33800 is a frameshifted gene for which two peptides could still be predicted.
