# Supplementary material for: Genome sequence of Xanthomonas fuscans subsp. fuscans strain 4834-R reveals that flagellar motility is not a general feature of xanthomonads
Source: BMC Genomics. 2013 Nov 6;14:761. doi: 10.1186/1471-2164-14-761 (PMC3826837; doi:10.1186/1471-2164-14-761)
Supplement: Additional file 9 — Primers for verification of frameshifts of selected CDSs. [file 1471-2164-14-761-S9.docx]

**Additional file 9. Primers used to amplify frameshifts detected by frameD.**

| Frameshifted  genes | primer name | Primer  sequences | fragment size (bp) | Annealing temperature |
| --- | --- | --- | --- | --- |
| XFF4834R_chr19550 | 5175-F | AATCGGCTTTGAAGGATGTGCC | 623 | 55°C |
|  | 5175-R | TCATAGCGCAAGAAGTTGATCC |  |  |
| XFF4834R_chr32500 | 5195-F | ATGAACACACTACGCGCTGTG | 342 | 55°C |
|  | 5195-R | TCCAGCGTGCCGTACGTGAAGT |  |  |
| XFF4834R_chr12700 | pilQ-F | GCTCAACGAGCTAACGAAAT | 684 | 55°C |
|  | pilQ-R | CAGCACCAGCAACACGCAAC |  |  |
| XFF4834R_chr26090 | 5085-F | GATATCGAGGCCTTGCGTCAG | 399 | 55°C |
|  | 5085-R | CCATGCATGCCGATACACATC |  |  |
| XFF4834R_chr34200 | xagA-F | CGCTGCAGTCCAGCTCGGTTG | 860 | 55°C |
|  | xagA-R | GCAACTTGGTGAGATCGTAGT |  |  |
| XFF4834R_chr40530 | 5143-F | GCTGGTGCGCACCCAATCGGA | 666 | 60°C |
|  | 5143-R | TGGCCTGCATCGACCGAATAC |  |  |
| XFF4834R_chr41250 | 5144-F | CTGGAACGCCGACGTTCCCGAAA | 467 | 60°C |
|  | 5144-R | AGATACTGTTTGGCAGCGGCCGT |  |  |
| XFF4834R_chr42820 | 5146-F | GCCTTGCGGGGGTACCTCTCGC | 369 | 60°C |
|  | 5146-R | CGCCATCTGCACCAACACCTCG |  |  |
| XFF4834R_chr15520 | 5170-F | CGCGGCTGGCAGGTGCACGGCGT | 545 | 60°C |
|  | 5170-R | CGATCAACACCGAAGGGCCAGC |  |  |
| XFF4834R_chr32600 | 5196-F | TCGATTACCAAGGCGACGATGGT | 443 | 60°C |
|  | 5196-R | CCAGCCAGGCGCTGCCATCGTCA |  |  |
| XFF4834R_chr36560 | 5201-F | CGCAACGTGGCCGCGCTGGACAAG | 423 | 60°C |
|  | 5201-R | CCGATGCATACCACCGCACCGACC |  |  |
| XFF4834R_chr41020 | 5210-F | TGGCAAACGTGTTTCGTCCG | 366 | 60°C |
|  | 5210-R | CCATCCAGCGGCACGAACTC |  |  |
| XFF4834R_chr20360 | 5127-F | CTGGATGCTGCCGGTGTGGCG | 563 | 60°C |
|  | 5127-R | TCCAGGCGGAACACTGCGACC |  |  |
| XFF4834R_chr09400 | 5084-F | GGCTGGGCGACCCAGGTGCCGA | 398 | 60°C |
|  | 5084-R | CCTGATTGGCGGCGGTGTTGTT |  |  |
